# PromptSTG: prototype-guided prompting for few-shot spatial transcriptomics annotation

**DOI:** 10.1093/bib/bbag401

**Published:** 2026-07-29

**Authors:** Renchu Guan, Ji Qi, Xueting Wang, Chuyao Wang, Yonghao Liu, Xiaoyue Feng, Lu Cui, Xiaosong Han

**Affiliations:** Key Laboratory of Symbolic Computation and Knowledge Engineering of the Ministry of Education, College of Computer Science and Technology, Jilin University, No. 2699 Qianjin Street, Changchun, Jilin Province, 130012, China; Key Laboratory of Symbolic Computation and Knowledge Engineering of the Ministry of Education, College of Computer Science and Technology, Jilin University, No. 2699 Qianjin Street, Changchun, Jilin Province, 130012, China; Key Laboratory of Symbolic Computation and Knowledge Engineering of the Ministry of Education, College of Computer Science and Technology, Jilin University, No. 2699 Qianjin Street, Changchun, Jilin Province, 130012, China; Key Laboratory of Symbolic Computation and Knowledge Engineering of the Ministry of Education, College of Computer Science and Technology, Jilin University, No. 2699 Qianjin Street, Changchun, Jilin Province, 130012, China; Key Laboratory of Symbolic Computation and Knowledge Engineering of the Ministry of Education, College of Computer Science and Technology, Jilin University, No. 2699 Qianjin Street, Changchun, Jilin Province, 130012, China; Key Laboratory of Symbolic Computation and Knowledge Engineering of the Ministry of Education, College of Computer Science and Technology, Jilin University, No. 2699 Qianjin Street, Changchun, Jilin Province, 130012, China; China-Japan Union Hospital of Jilin University, No. 126 Xiantai Street, Changchun, Jilin Province, 130033, China; Key Laboratory of Symbolic Computation and Knowledge Engineering of the Ministry of Education, College of Computer Science and Technology, Jilin University, No. 2699 Qianjin Street, Changchun, Jilin Province, 130012, China

**Keywords:** spatial transcriptomics, cell type annotation, graph neural networks, few-shot learning

## Abstract

Single-cell resolution spatial transcriptomics (scST) simultaneously captures gene expression and spatial coordinates at an unprecedented scale, which provides powerful opportunities to dissect tissue architecture and cell-cell interactions. However, accurate cell type annotation remains challenging, particularly in scenarios where reliable labels are scarce and manual annotation is costly. These challenges are further amplified in tissues characterized by complex spatial dependencies and pronounced cellular heterogeneity, especially within tumor microenvironments. To address these issues, we propose Prompt-guided Spatial Transcriptomics Graph (PromptSTG), a graph-based few-shot learning framework for robust cell type annotation in scST data. PromptSTG integrates spatial information and transcriptomic features to model biologically coherent cellular neighborhoods and enables accurate label propagation from a small set of labeled cells to large unlabeled populations. Across extensive benchmarks spanning multiple spatial transcriptomics platforms and tissue types, PromptSTG consistently outperforms existing methods in annotation accuracy, robustness, and scalability under few-shot settings. Moreover, PromptSTG reconstructs spatially coherent tissue organization and effectively identifies rare yet biologically important cell populations, such as immature oligodendrocytes and ependymal cells, which together account for less than 7% of total cells. In complex tissue environments, the method preserves both global tissue structure and fine-grained cellular boundaries, yielding biologically meaningful and spatially consistent annotations. All source codes are available at https://github.com/KEAML-JLU/PromptSTG.

## Introduction

Understanding cellular composition and molecular heterogeneity within tissues is a central goal of transcriptomic research [1–3]. Spatial transcriptomics (ST) technologies enable the simultaneous measurement of gene expression and spatial location within intact tissue sections [4, 5], allowing transcriptional programs to be analyzed in their anatomical context and providing powerful tools for investigating tissue organization in development and disease [6]. With the emergence of high-throughput ST platforms such as MERFISH [7], Stereo-seq [8, 9], and Xenium [10], ST data have reached single-cell resolution [11]. However, due to uneven cell distribution and pronounced cellular heterogeneity within tissues, accurate cell type annotation at the whole-slide level remains challenging [12, 13].

Substantial progress has been made in cell type annotation for ST. scmap [14] relies on transcriptomic similarity to project ST data onto reference single-cell datasets. Cell2location [15] employs a Bayesian framework to integrate single-cell reference data with ST, enabling the inference of cell-type abundances at each spatial location. Tangram [16] uses an optimization-based alignment strategy to map single-cell expression patterns onto spatial transcriptomic data while preserving cell-type information. jMF2D [17] leverages joint non-negative matrix factorization and a cell-type similarity network to achieve efficient integration of single-cell RNA-seq and ST data. Methods such as scPoli [18] and DSCT [19] employ deep learning strategies, including semi-supervised and contrastive learning, to improve cross-dataset transferability. Spatial-ID [20] explicitly incorporates spatial information into the annotation process.

Despite substantial progress in cell type annotation for ST in recent years, most existing methods still rely on the assumption that cross-modal data can be aligned in a shared latent space, as well as on fixed representation learning paradigms [21–23]. However, the lack of well-matched single-cell reference data for many ST datasets undermines the effectiveness of cross-modal alignment. In addition, manual annotation of ST data is costly and prone to subjective bias [24, 25], leading to a scarcity of labeled data [26–28]. As a result, these methods still suffer from constrained generalization ability in such scenarios [29–31].

To address these challenges, we propose **PromptSTG** (**Prompt**-guided **S**patial **T**ranscriptomics **G**raph), a few-shot annotation framework for single-cell resolution spatial transcriptomics (scST) data. PromptSTG integrates graph representation learning with prompt-based adaptation to enable accurate cell type prediction across whole tissue sections under minimal supervision. The main contributions of this work are summarized as follows:

**(1) A few-shot learning-based annotation framework for scST data.** To tackle the challenge of sparse supervision in scST data, we cast cell type annotation as a few-shot learning problem and propose a prototype-guided framework. Specifically, we devise two complementary loss terms, namely a prototype separation loss and a compactness loss, that jointly promote inter-class distinctness and intra-class cohesion, ultimately boosting discriminative power and generalization under label-scarce conditions.

**(2) Soft attention-based prompt mechanism.** We introduce learnable task-level and class-level soft prompts that modulate graph representation learning. These prompts allow sparse labels to explicitly guide representation refinement and decision boundary formation, enabling the model to adapt to specific annotation tasks without extensive retraining.

**(3) Multi-relational graph fusion modeling.** We propose a multi-relational graph fusion strategy that jointly models spatial proximity and gene expression similarity. This integration mitigates the interference of heterogeneous cellular relationships in complex tissue environments and enhances the structural consistency and biological relevance of cell representations, providing a robust foundation for downstream annotation.

Through extensive experiments on diverse datasets, PromptSTG demonstrates superior accuracy, robustness, and scalability, consistently outperforming existing methods under challenging few-shot annotation settings.

## Materials and methods

### PromptSTG workflow

**Figure 1.**
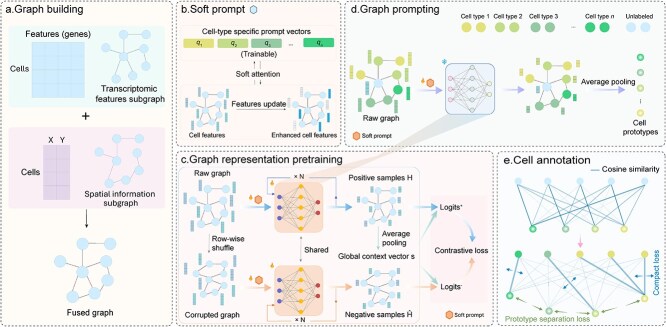
Overview of the PromptSTG framework. (a) Graph building. Edges in the cell expression subgraph are constructed by connecting each cell to its k nearest neighbors in transcriptomic space, while edges in the spatial information subgraph are defined based on spatial proximity using KNNs or radius-based criteria. The two subgraphs are fused to form a unified cell graph that integrates molecular similarity and spatial adjacency. (b) Soft prompt. A set of learnable prompt vectors supplements the feature vectors via a soft attention mechanism. (c) Graph representation pretraining. A graph contrastive learning strategy is used to pretrain the graph encoder. The model learns to maximize mutual information between node embeddings and the global context vector, optimized via contrastive loss. (d) Graph prompting. The pretrained graph encoder is frozen, and the prompt vectors are fine-tuned using limited labeled data. Initial cell prototypes for each cell type are established through average pooling on the labeled data. (e) Cell annotation. The compact loss pulls embeddings toward their corresponding prototypes, and the prototype separation loss enlarges inter-type distances. Cell types are predicted by computing cosine similarity between each embedding and the prototypes.

The overall framework of PromptSTG is illustrated in Fig. 1, which consists of four key steps: graph building, graph representation pretraining, graph prompting, and cell annotation. We formulate the problem as a transductive learning task, where the graph is constructed over all nodes from the whole tissue section during pretraining, while only a small subset of labeled nodes is used for downstream generalization.

PromptSTG begins by preprocessing the ST data, removing cells with ambiguous labels. A graph is then constructed by integrating spatial proximity and gene expression similarity, where each cell is treated as a node and its processed gene expression profile serves as node features. Specifically, adjacency relationships are first determined based on spatial neighborhoods and further refined using gene expression similarity to better capture cellular relationships in heterogeneous tissues. The final graph structure is obtained by fusing the spatial and expression adjacency matrices (Fig. 1a).

To enhance model adaptability, PromptSTG introduces learnable global prompt vectors to each node through a soft attention mechanism (Fig. 1b). During the graph representation learning stage, a graph convolutional network (GCN) [32] is employed to perform self-supervised contrastive learning, enabling the model to learn robust cell embeddings (Fig. 1c). In the subsequent graph prompting stage, only parameters associated with the prompt vectors are fine-tuned, while the pretrained backbone remains fixed. In the cell type annotation stage, we first perform few-shot sampling for each class and initialize class prototypes by applying average pooling over the labeled samples (Fig. 1d). In the downstream annotation task, a prototype-guided strategy is adopted. Cell embeddings are encouraged to approach their corresponding prototypes via a compact loss, while inter-prototype discrimination is enhanced using a prototype separation loss. Finally, cell types are assigned based on cosine similarity between cell embeddings and prototypes (Fig. 1e).

### Graph building

#### Spatial information subgraph

We first constructed a subgraph structure based on the spatial locations of the cells. Specifically, we applied the K-nearest neighbors (KNN) algorithm to identify the 20 nearest neighbors for each cell using Euclidean distance and assigned edge weights according to a Gaussian kernel function. Formally, the spatial information subgraph is calculated as Equation (1): 


(1) \begin{align*}& \mathrm{A}^{(1)}_{ij} = \begin{cases} \exp\!\left( -\dfrac{\lVert \mathrm{\mathrm{s}}_{i} - \mathrm{\mathrm{s}}_{j} \rVert^{2}} {2\,\mathrm{\sigma}^{2}} \right), & j \in \mathcal{N}(i), \\[8pt] 0, & j \notin \mathcal{N}(i), \end{cases}\end{align*}

where $\mathrm{s}_{i}$ and $\mathrm{s}_{j}$ denote the spatial coordinates of cells $i$ and $j$, and $\sigma $ is a scaling parameter, with a default value of $0.01$. $\mathcal{N}(i)$ denotes the set of neighbors of cell $i$.

The resulting raw adjacency matrix $\mathrm{A}^{(1)}$ was normalized using Equation (2): 


(2) \begin{align*}& \mathrm{A}^{(1)} = \mathrm{D}^{-\frac{1}{2}} \mathrm{A}^{(1)} \mathrm{D}^{-\frac{1}{2}},\end{align*}

where $\mathrm{D}$ is the corresponding degree matrix.

To evaluate the structural properties of the spatial graph, we computed its edge heterogeneity. The calculation of edge heterogeneity is given in Equation (3): 


(3) \begin{align*} &dh_{\mathrm{edge}}^{\mathrm{expr}} = \frac{\sum_{(i,j)\in E} \mathrm{A}^{(1)}_{ij} \cdot \left(1 - \cos(\mathrm{x}_{i}, \mathrm{x}_{j})\right)} {\sum_{(i,j)\in E} \mathrm{A}^{(1)}_{ij}},\nonumber\\ &\qquad\quad \cos(\mathrm{x}_{i}, \mathrm{x}_{j}) = \frac{\mathrm{x}_{i} \mathrm{x}_{j}^{\top}} {\|\mathrm{x}_{i}\|_{2} \, \|\mathrm{x}_{j}\|_{2}},\end{align*}

where $E$ is the edge set of the graph, and $(i,j)$ denotes an edge between cells $i$ and $j$. $\mathrm{x}_{i}$ and $\mathrm{x}_{j}$ represent the preprocessed gene expression profiles of cells $i$ and $j$. Detailed results of this calculation are presented and discussed in the Supplementary Graph fusion Section.

#### Cell expression subgraph

To mitigate the high heterophily observed in the spatial information subgraphs, we further constructed a cell expression subgraph. We used the preprocessed gene expression matrix $\mathrm{X} \in \mathbb{R}^{N \times d}$, where $N$ is the number of cells and $d$ is the feature dimensionality. In all cases, we employed the KNN algorithm with cosine similarity to identify the neighbor set of each cell. Formally, the cell expression subgraph is calculated as Equation (4): 


(4) \begin{align*}& \mathrm{A}_{ij}^{(2)} = \begin{cases} \cos(\mathrm{x}_{i}, \mathrm{x}_{j}), & j \in \mathcal{N}(i), \\[8pt] 0, & j \notin \mathcal{N}(i), \end{cases}\end{align*}

where $\mathrm{x}_{i}$ and $\mathrm{x}_{j}$ represent the preprocessed gene expression profiles of cells $i$ and $j$. $\mathcal{N}(i)$ denotes the set of neighbors of cell $i$.

#### Graph fusion

We designed two graph fusion strategies to integrate spatial and gene expression subgraphs.

**(1) Weak-link removal fusion**

The first is weak-link removal fusion. In this strategy, a relatively large threshold $\alpha $ is introduced during graph fusion to penalize spatial connections that are weakly supported by gene expression similarity. As a result, cells that are spatially adjacent but exhibit substantially different gene expression profiles receive reduced edge weights or are removed entirely. The weak-link removal fusion strategy is defined in Equation (5): 


(5) \begin{align*}& \hat{\mathrm{A}} = \max\!\left( \mathrm{A}^{(1)} + \mathrm{A}^{(2)} - \alpha,\ 0 \right).\end{align*}

**(2) Normalized fusion**

The second is normalized fusion. In this strategy, both subgraphs are degree-normalized. The subgraphs are then summed and further normalized. The normalized fusion strategy is defined in Equation (6): 


(6) \begin{align*} &\qquad \ \ \ \mathrm{A}^{(*)} = \mathrm{D}^{-\frac{1}{2}} \mathrm{A}^{(*)} \mathrm{D}^{-\frac{1}{2}},\nonumber\\ &\hat{\mathrm{A}} = \mathrm{Normalize}_{L_{1}}\!\left(\mathrm{A}^{(1)} + \mathrm{A}^{(2)} + \mathbb{I}\right),\end{align*}

where * denotes either 1 or 2, $D$ denotes the corresponding degree matrix, and $\mathrm{Normalize}_{L_{1}}(\cdot )$ denotes row-wise $L_{1}$ normalization to ensure that the sum of each row equals 1. $\mathbb{I}$ is the identity matrix that adds a self-loop to each node. We specifically discuss the design motivations and application scenarios of these two graph fusion strategies in the Supplementary Graph fusion section.

### Soft prompt

We introduced a global prompt matrix $\mathrm{Q} \in \mathbb{R}^{C \times d}$ to supplement the feature vectors. Specifically, for each cell type $c$, we defined a trainable prompt vector $\mathrm{q}_{c} \in \mathbb{R}^{d}$ that shares the same dimensionality as the feature vectors. We computed soft attention scores between each feature vector and all type-specific prompt vectors. The process can be defined by Equation (7): 


(7) \begin{align*} &\qquad\quad\qquad\mathrm{Z} = \mathrm{X} \mathrm{W} + \mathrm{b},\nonumber\\ \mathrm{S}_{i,c} = &\frac{\exp(\mathrm{Z}_{i,c})}{\sum_{k=1}^{C} \exp(\mathrm{Z}_{i,k})}, \quad i = 1,\dots,N,\; c = 1,\dots,C,\\ &\qquad\quad\qquad\mathrm{X} = \mathrm{X} + \mathrm{SQ}\nonumber\end{align*}

where $\mathrm{W}$ is the trainable weight matrix of the linear layer, $\mathrm{b}$ is the corresponding bias vector, $\mathrm{S} \in \mathbb{R}^{N \times C}$ contains the attention scores for all cells, and $\mathrm{SQ}$ denotes the prompt-enhanced feature vector for each cell, obtained by aggregating the prompt matrix $\mathrm{Q}$ according to the attention scores in $\mathrm{S}$.

### Graph representation pretraining

Recent studies have shown that pretraining enables models to learn more generalizable knowledge [33, 34]. Here, we adopted the contrastive pretraining framework—deep graph infomax (DGI) [35] to learn node representations in a self-supervised manner. Specifically, we used a single-layer GCN to aggregate information from both the node itself and its neighbors. The GCN propagation rule is defined in Equation (8): 


(8) \begin{align*} &\operatorname{ELU}(x) = \begin{cases} x, & x> 0, \\ e^{x} - 1, & x \le 0, \end{cases}\\ &\qquad\mathrm{H} = \operatorname{ELU}(\hat{\mathrm{A}} \mathrm{X} \mathrm{W}),\nonumber\end{align*}

where $\operatorname{ELU}(\cdot )$ denotes the exponential linear unit (ELU) activation function (the comparison and selection of activation functions are discussed in the Supplementary Activation Function Section), $\hat{\mathrm{A}}$ is the fused adjacency matrix, $\mathrm{W}$ is the learnable parameter matrix, and $\mathrm{H}$ represents the positive sample embeddings.

Negative samples were generated by corrupting the input feature matrix through random row-wise shuffling, resulting in $\hat{\mathrm{X}}$. The same GCN was then applied to $\hat{\mathrm{X}}$ to obtain the negative sample embeddings $\hat{\mathrm{H}}$. The computation of the negative sample embeddings is given in Equation (9): 


(9) \begin{align*}& \hat{\mathrm{H}} = \mathrm{ELU}\!\left(\hat{\mathrm{A}}\ \hat{\mathrm{X}}\mathrm{W}\right).\end{align*}

Then, a global context vector $\mathrm{s}$ was computed by applying average pooling over the positive sample embeddings $\mathrm{H}$, which is defined as: 


(10) \begin{align*} &\mathrm{s} = \sigma\!\left(\frac{1}{N}\sum_{i=1}^{N} \mathrm{h}_{i} \right),\nonumber\\ &\ \ \ \sigma(x) = \frac{1}{1 + e^{-x}},\end{align*}

where $\mathrm{h}_{i}$ denotes the embedding of the positive samples of node $i$.

Finally, a binary cross-entropy contrastive loss was used to maximize mutual information between the node embeddings and the global context vector. The binary cross-entropy contrastive loss is given in Equation (11): 


(11) \begin{align*} &\qquad\qquad\qquad\quad s_{i}^{+} = \mathrm{h}_{i} \mathrm{W} \mathrm{s}^\top,\nonumber\\ &\qquad\qquad\qquad\quad s_{i}^{-} = \hat{\mathrm{h}}_{i} \mathrm{W} \mathrm{s}^\top,\\ &\mathcal{L}_{DGI} = - \sum_{i=1}^{N} \Big[ \log \sigma(s_{i}^{+}) + \log (1 - \sigma(s_{i}^{-})) \Big],\nonumber\end{align*}

where $\hat{\mathrm{h}}_{i}$ denotes the embedding of the negative samples of node $i$, $\mathrm{W}_{\mathrm{s}}$ is the trainable bilinear weight matrix.

### Graph prompting and cell annotation

To mitigate overfitting in the downstream task, the DGI-related parameters were frozen, and only the prompt vectors were fine-tuned using labeled data under a few-shot setting. We set the cell prototypes as trainable tensors and applied average pooling on the labeled data to establish an initial prototype $\mathrm{p}_{c}$ for each cell type: 


(12) \begin{align*} &I_{c} = \{\, i \mid y_{i} = c \,\},\nonumber\\ &\mathrm{p}_{c} = \frac{1}{|I_{c}|} \sum_{i \in I_{c}} \mathrm{h}_{i},\end{align*}

where $I_{c}$ is the set of cells belonging to cell type $c$, $|I_{c}|$ is the number of cells in set $I_{c}$.

We determined the cell types by computing the cosine similarity between each cell and the cell prototypes. The decision-making process is defined in Equation (13): 


(13) \begin{align*} &z_{i,c} = \frac{\cos(\mathrm{h}_{i}, \mathrm{p}_{c})}{\tau},\nonumber\\ &\hat{y}_{i} = \arg\max_{c} z_{i,c},\end{align*}

where $\tau $ is the temperature parameter, which controls the sharpness of the similarity distribution. $\hat{y}_{i}$ is the predicted cell type of cell $i$.

We used the cross-entropy loss to measure the discrepancy between the similarity matrix and the true labels. The cross-entropy loss can be defined by Equation (14): 


(14) \begin{align*} &\qquad\hat{p}_{i,c} = \frac{e^{z_{i,c}}}{\sum_{j=1}^{C} e^{z_{i,j}}},\nonumber\\ &\mathcal{L}_{\mathrm{CE}} = -\frac{1}{N_{\mathrm{train}}} \sum_{i=1}^{N_{\mathrm{train}}} \log \hat{p}_{i,y_{i}},\end{align*}

where $N_{\mathrm{train}}$ is the number of labeled cells.

At the same time, we used the compact loss to pull node embeddings closer to their corresponding prototypes, while the prototype separation loss increased the distance between different cell prototypes. The above losses are given in Equation (15): 


(15) \begin{align*} &\qquad\qquad\mathcal{L}_{\mathrm{compact}} = \frac{1}{N_{\mathrm{train}}} \sum_{i=1}^{N_{\mathrm{train}}} \Big[ 1 - \cos(\mathrm{h}_{i}, \mathrm{p}_{y_{i}}) \Big],\nonumber\\ &\mathcal{L}_{\mathrm{sep}} = \frac{1}{2 C (C - 1)} \sum_{i \neq j} \max \Bigg(0, \beta - \Big\| \frac{\mathrm{p}_{i}}{\|\mathrm{p}_{i}\|_{2}} - \frac{\mathrm{p}_{j}}{\|\mathrm{p}_{j}\|_{2}} \Big\|_{2} \Bigg),\end{align*}

where $\beta $ is a hyperparameter that controls the minimum distance between cell prototypes.

The total loss can be defined by Equation (16): 


(16) \begin{align*}& \mathcal{L}_{\mathrm{total}} = \mathcal{L}_{\mathrm{CE}} + \mathcal{L}_{\mathrm{compact}} + \mathcal{L}_{\mathrm{sep}}.\end{align*}

## Results

### Datasets

To comprehensively evaluate the performance of PromptSTG, we selected three scST datasets generated using different experimental platforms, covering various tissue types, technical resolutions, and molecular characteristics.

The MERFISH mouse hypothalamus dataset [36] comprises $\sim $one million cells from the preoptic area of the mouse hypothalamus, organized into 36 tissue sections. Each cell is associated with precise spatial coordinates and rich metadata. This dataset features ultra-high cell density and complex neuroanatomical structures, making it suitable for evaluating model performance on high-resolution spatial data, while biological variability among individual mice provides a basis for evaluating model robustness.

The human colorectal adenocarcinoma (COAD) dataset [37] was generated using the Stereo-seq v1.3 platform from treatment-naïve tumor tissue, providing subcellular spatial resolution ($\le $ 2 $\mu $m) and transcriptomic profiles covering 5001–18 085 genes per cell, with cell type annotation obtained by integrating single-cell RNA sequencing (scRNA-seq) and proteomics data. Its pronounced tumor microenvironment heterogeneity and complex tissue architecture make it appropriate for evaluating the model’s ability to resolve fine-grained cell types, characterize spatial organization, and generalize to large-scale cellular data.

The breast cancer Xenium dataset [38] is derived from FFPE human breast tumor samples. We used Xenium In Situ data from one patient sample, including both ductal carcinoma in situ and invasive ductal carcinoma regions. This dataset provides subcellular-resolution expression profiles based on a targeted gene panel and is linked to whole-transcriptome scRNA-seq and ST data. Variations in histopathological state, receptor expression, and technical modality increase biological and technical complexity, making it suitable for evaluating the model’s adaptability and robustness in clinically complex tissue environments.

To reduce the effects of technical noise, extreme expression variability, and low-quality cells, a unified standardized preprocessing pipeline was applied to all datasets. Cells with ambiguous or unreliable labels were first removed. The mouse hypothalamus dataset was divided into 36 subsets according to Animal-ID, and each subset was analyzed independently. For the COAD dataset, cells with total mRNA counts < 50 were filtered out, followed by library-size normalization, log1p transformation, highly variable gene selection, and feature scaling. For the breast cancer Xenium dataset, after removing low-quality cells, the expression matrix was normalized, and principal component analysis was performed, with the top 50 principal components retained as transcriptomic feature representations. All processed data were stored in AnnData format for downstream modeling. The number of cells and genes in the processed datasets are provided in Supplementary Table 2.

### Accurate annotation of rare neural cell types in mouse hypothalamus

**Figure 2.**
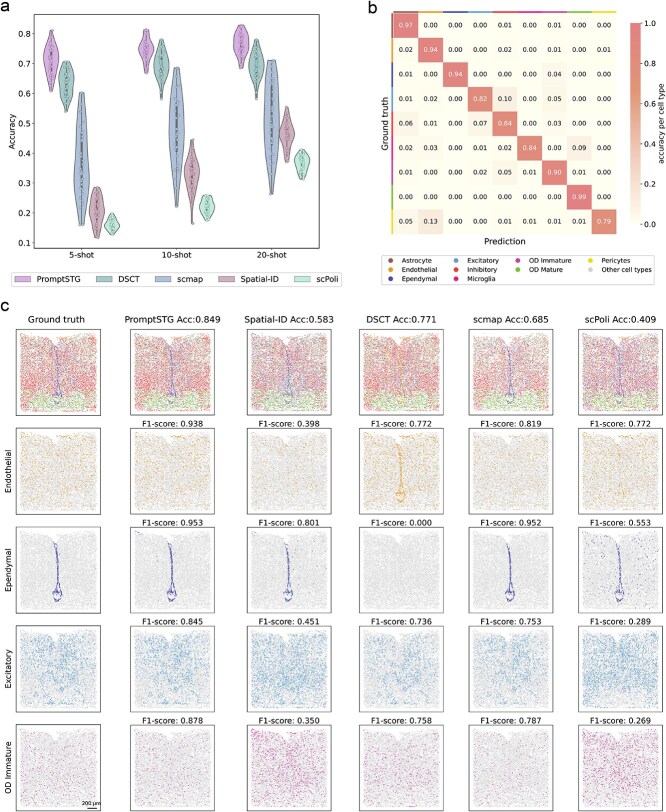
Benchmarking of PromptSTG against existing annotation methods in mouse hypothalamus ST data. (a) The violin plots illustrate the distribution of prediction accuracy for PromptSTG and baseline methods under 5-shot, 10-shot, and 20-shot settings across 36 Animal-ID sections, with the width of each violin representing the density of accuracy values. (b) Confusion matrix showing prediction accuracy across cell types (Animal-ID: 1). (c) Visualization of cell type annotation results projected onto the spatial coordinates of mouse hypothalamus slices (Animal-ID: 1). Ground truth and predictions from different methods are shown side by side. Rows correspond to representative cell types (endothelial, ependymal, excitatory, OD immature), with F1-scores reported for each method. Each subpanel displays only one of the above cell types, while the other cell types are shown in gray.

To evaluate the performance of PromptSTG, we conducted a benchmarking analysis on all 36 subsets generated by MERFISH (Supplementary Table 3). Across the 5-shot, 10-shot, and 20-shot settings, PromptSTG consistently achieved the highest mean accuracy (0.711, 0.748, and 0.764, respectively) compared with baseline methods (Fig. 2a and Supplementary Table 3). It also exhibited low coefficients of variation (0.066, 0.050, and 0.048), indicating high stability. In the most challenging 5-shot setting, PromptSTG substantially outperformed all competitors, with DSCT reaching 0.635 accuracy and the remaining methods performing far worse. This advantage was maintained across all subsets and became more pronounced as the number of labeled cells increased, underscoring the robustness and scalability of PromptSTG. Notably, for certain rare cell types, some baseline methods—particularly scmap—also achieved reasonable accuracy, suggesting that clustering-based matching in a well-preprocessed cell expression space still provides discriminatory power. These observations highlight that PromptSTG’s architectural components work synergistically to deliver high and stable performance, particularly in the few-shot regime.

We next evaluated the cell type-specific prediction performance of PromptSTG relative to benchmarking methods in the 5-shot setting (Fig. 2b and Supplementary Fig. S2). PromptSTG achieved high accuracy for major neural populations, including astrocytes, excitatory neurons, and mature oligodendrocytes. Importantly, it also showed superior performance on ependymal cells, which represent only about 2.3% of the dataset. PromptSTG reached an accuracy of 0.937, surpassing scmap (0.862). By contrast, DSCT and Spatial-ID failed to correctly annotate this population (accuracy = 0), underscoring the challenge of annotating low-abundance cell types.

Finally, we evaluated the spatial distribution of predicted cell types under the 20-shot setting (Fig. 2c and Supplementary Fig. S3). Compared to baseline methods, PromptSTG better preserves fine-scale tissue architecture as well as global spatial organization. In particular, PromptSTG demonstrates improved reliability in identifying both rare and abundant cell populations. For instance, immature oligodendrocytes ($\sim $4% of cells) and ependymal cells ($\sim $2%) are accurately recovered alongside major populations such as endothelial cells and excitatory neurons.

To further validate the biological plausibility of these rare cell types, we examined the expression of canonical marker genes, *Pdgfra* for immature oligodendrocytes [39] and *Cd24a* for ependymal cells [40], and compared their enrichment patterns between ground-truth annotations and model predictions (Supplementary Fig. S4). In the ground-truth labels, *Cd24a* is highly expressed in ependymal cells relative to other cell types (28.94 versus 1.40), while *Pdgfra* shows strong enrichment in immature oligodendrocytes (44.26 versus 0.53). Notably, this cell type-specific enrichment is well preserved in the predicted results, with *Cd24a* (27.02 versus 1.40) and *Pdgfra* (44.26 versus 0.59) showing higher expression in their corresponding cell populations.

Together, these results indicate that PromptSTG not only captures coherent spatial organization but also preserves biologically meaningful molecular signatures, supporting the validity of the predicted cell type annotations.

### Superior performance on a large-scale Stereo-seq COAD dataset

**Figure 3.**
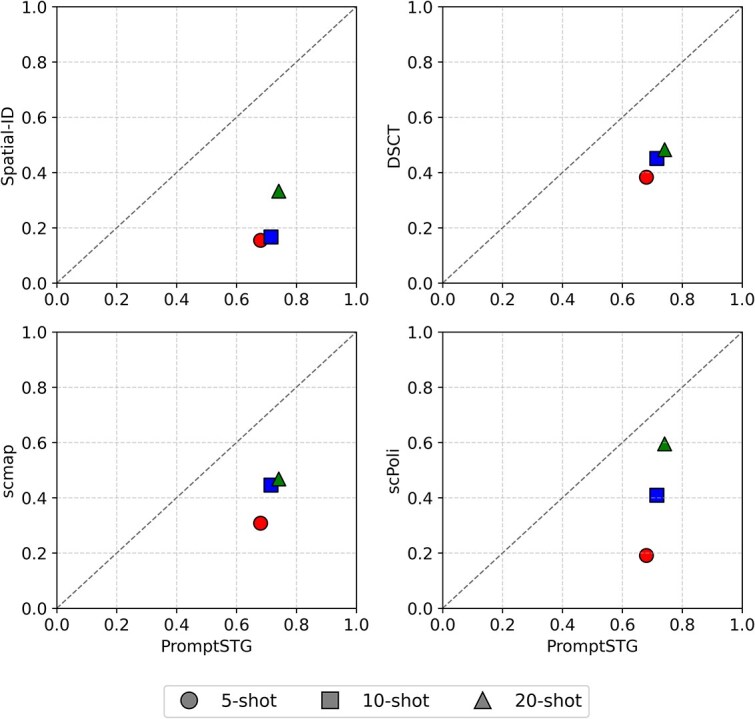
Comparative accuracy in Stereo-seq COAD data: each point compares PromptSTG against a benchmark method under 5-shot (circles), 10-shot (squares), or 20-shot (triangles), with x and y coordinates representing the accuracies of PromptSTG and the benchmark method (Spatial-ID, DSCT, scmap, or scPoli), respectively, such that points below the diagonal (y < x) favor PromptSTG, while scPoli results under the 5-shot setting are omitted due to insufficient labeled samples.

**Figure 4.**
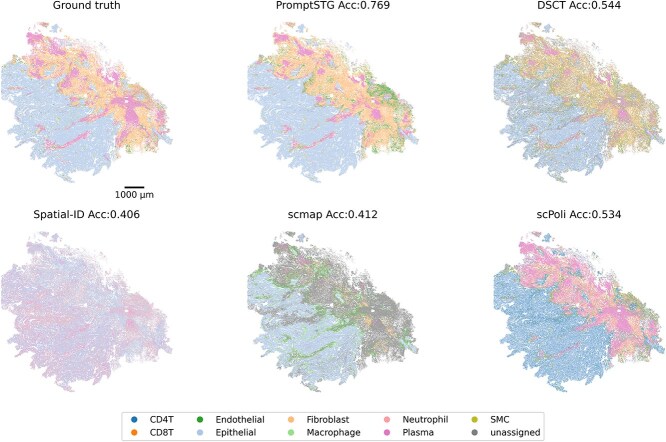
Spatial visualization of cell type annotation in Stereo-seq COAD data, with PromptSTG (Acc = 0.769) demonstrating improved spatial structure recovery compared to baseline methods.

Building on the robust performance of PromptSTG in the mouse hypothalamus dataset, we next evaluated the method on the COAD dataset generated using the sequencing-based ST technology Stereo-seq. Global annotation accuracy across different supervision levels is summarized in Fig. 3 and Supplementary Table 4. PromptSTG consistently outperformed all benchmarking methods under few-shot settings. Spatial visualization of cell type annotation under the 20-shot setting is shown in Fig. 4 and Supplementary Fig. S5. Under this setting, PromptSTG achieved an overall accuracy of 0.741, substantially higher than DSCT (0.483), scmap (0.468), Cell2location (0.330), Tangram (0.150), jMF2D (0.066), Spatial-ID (0.333), and scPoli (0.595).

As illustrated in Fig. 4, PromptSTG produces spatial patterns that closely recapitulate the ground truth, with clear and coherent delineation of major tumor-associated compartments, including epithelial tumor regions, immune infiltrates (CD4T, CD8T, macrophages, neutrophils, and plasma cells), as well as stromal components such as fibroblasts, endothelial cells, and smooth muscle cells. Importantly, PromptSTG preserves sharp boundaries at tumor-stromal and tumor-immune interfaces, while maintaining spatial continuity within each population.

In contrast, competing methods exhibit pronounced deficiencies in resolving the complex tumor microenvironment. Spatial-ID produces highly fragmented and noisy spatial patterns, obscuring biologically meaningful tissue organization. DSCT and scmap show blurred or incomplete boundaries, particularly in regions where immune and stromal cells are spatially intermingled with epithelial tumor cells. scPoli captures coarse global trends but fails to accurately delineate fine-scale spatial structures and mixed cellular neighborhoods.

These qualitative observations are consistent with the quantitative accuracy metrics and demonstrate that PromptSTG effectively captures both global tissue architecture and fine-grained spatial heterogeneity in large-scale and noisy Stereo-seq COAD data. By faithfully reconstructing spatially coherent cell distributions across tumor, immune, and stromal compartments, PromptSTG provides reliable cell type annotation in highly heterogeneous cancer microenvironments.

### Robust annotation and biological interpretation of human breast cancer Xenium data

**Figure 5.**
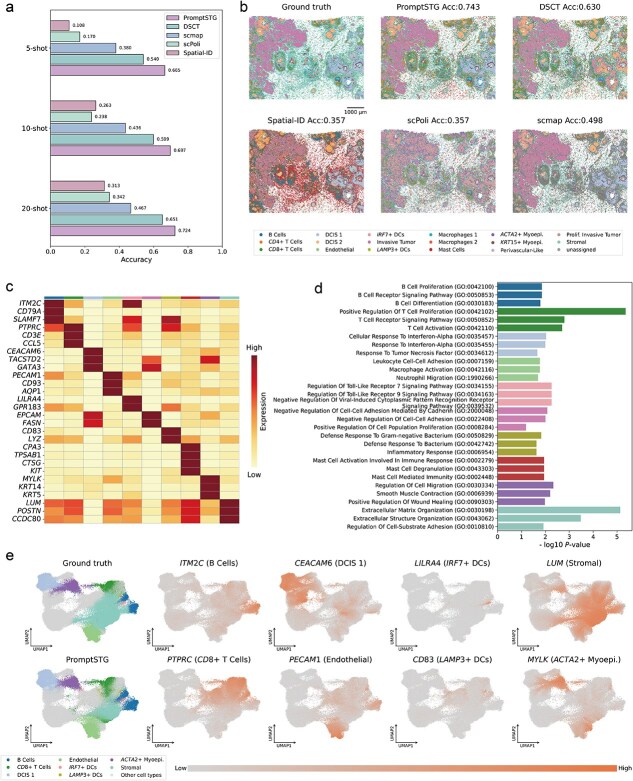
Analysis of Xenium human breast cancer data, with (a) accuracy comparison of cell type annotation across different methods, (b) spatial visualization of cell type annotation across different methods, (c) heatmap of cell type-specific marker expression derived from PromptSTG annotations, (d) GO enrichment analysis of marker genes identified from PromptSTG annotations performed using gseapy based on Fisher's Exact Test with FDR correction by the Benjamini-Hochberg method, and (e) UMAP visualization showing ground-truth cell type labels and PromptSTG annotations together with spatial expression patterns of selected marker genes.

To further evaluate the robustness and biological interpretability of PromptSTG, we applied it to human breast cancer data generated by Xenium. PromptSTG demonstrated clear quantitative advantages across all few-shot settings (Fig. 5a and Supplementary Table 5). Specifically, it achieved the highest accuracy in the 5-shot (0.665), 10-shot (0.697), and 20-shot (0.724) scenarios, substantially outperforming competing methods by large margins. In addition, its spatial annotation accurately reconstructed tumor cores, stromal regions, and immune infiltrates with well-preserved boundaries (Fig. 5b and Supplementary Fig. S6). Notably, PromptSTG recovered biologically meaningful but rare populations, including *IRF7+* and *LAMP3+* dendritic cells, which were frequently misannotated by other methods. These populations are sparsely distributed and spatially intermingled with other immune and tumor cells, making them particularly challenging to resolve in complex tumor tissues.

To further assess the biological validity of the predicted annotations, we performed differential expression analysis based on the cell type labels inferred by PromptSTG. Canonical markers showed clear and specific enrichment within the corresponding predicted populations, including *CD79A* in B cells, *PTPRC* in T cells, *PECAM1* in endothelial cells, and *EPCAM* in invasive tumor cells (Fig. 5c). Rare immune subsets also exhibited distinct marker signatures, such as *LILRA4* and *CD83* in *IRF7+* and *LAMP3+* dendritic cells, respectively, supporting the accuracy of PromptSTG in resolving minor but functionally important populations.

Gene ontology (GO) enrichment analysis was performed for each predicted cell type based on their differentially expressed genes, as shown in Fig. 5d. For example, *CD8+* T cells are enriched in pathways related to T cell receptor signaling, T cell activation, and proliferation, which are characteristic of activated cytotoxic T cell responses and are consistent with their role in immune surveillance and tumor cell killing. In contrast, stromal cells show strong enrichment in extracellular matrix organization, extracellular structure organization, and cell adhesion-related processes, indicating their involvement in maintaining tissue integrity and regulating microenvironmental remodeling. Similarly, B cells are enriched in B cell receptor signaling, proliferation, and differentiation pathways, consistent with active adaptive immune responses and antibody-mediated immunity.

Combined with the UMAP spatial visualization results (Fig. 5e), the spatial expression patterns of representative marker genes are highly consistent with the predicted distributions of their corresponding cell types, indicating that PromptSTG’s predictions are not only accurate but also preserve biologically meaningful spatial organization, effectively resolving the complex tumor microenvironment, which is characterized by diverse cell populations and spatially intermingled tumor-immune-stromal compartments.

### Parameter sensitivity and ablation analysis

To evaluate the robustness of PromptSTG to hyperparameter selection, we conducted a parameter sensitivity analysis on the mouse hypothalamus dataset, which contains 36 sections and enables a more comprehensive assessment. We report results under both validation-based early stopping and fixed-epoch training settings (see Supplementary Experimental setup section).

**Figure 6.**
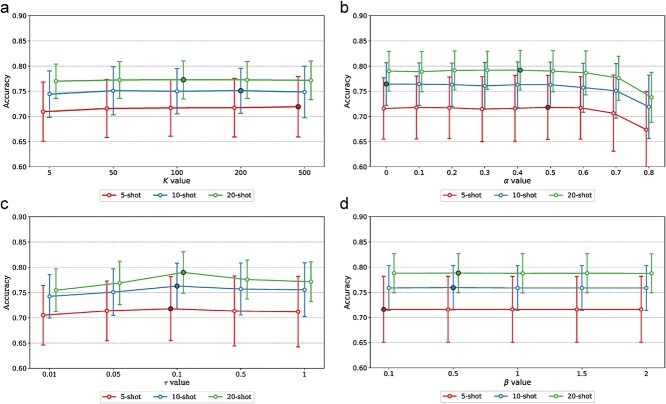
Evaluation of PromptSTG performance under parameter sensitivity analysis. (a) Parameter sensitivity analysis of the number of neighbors $K$ used in the cell expression subgraph under three few-shot settings. (b) Parameter sensitivity analysis of the graph fusion threshold $\alpha $ under three few-shot settings. (c) Parameter sensitivity analysis of the temperature parameter $\tau $ under three few-shot settings. (d) Parameter sensitivity analysis of the prototype distance threshold $\beta $ under three few-shot settings. The best performance under each setting is highlighted by a solid dot.

We first examined the effect of the number of neighbors $K$ in the cell expression subgraph (Fig. 6a). Across all few-shot settings, model performance gradually saturates as $K$ increases to 100–500, with larger labeled sets reaching saturation at smaller $K$ values. This trend is consistent with the heterophily analysis in the Graph fusion section of the Supplementary Materials, suggesting that incorporating sufficient expression-based neighbors helps reduce spatial heterogeneity and improves the capture of underlying biological structures. For example, at $K=200$, the edge heterophily is 0.115, supporting effective information integration. In contrast, smaller $K$ values (e.g. $K=50$) lead to inferior performance, likely due to insufficient expression-level context despite slightly lower heterogeneity. Under the fixed-epoch setting, the model achieves relatively strong performance at $K=5$, followed by a decrease around $K=50$, and then gradually recovers as $K$ further increases (Supplementary Fig. S7a). Combined with the stable results observed under early stopping, this non-monotonic behavior suggests a mismatch between neighborhood scale and the optimal training duration.

We next analyzed the threshold $\alpha $ used to remove weak links in the first graph fusion strategy (Fig. 6b). When $\alpha \leq 0.5$, accuracy remained stable at 0.717, 0.763, and 0.791 for the three shot settings, with standard deviations below 0.001. This stability indicates that a low threshold removes only a small number of noisy edges, so the fused graph topology remains largely dominated by the cell expression subgraph. Accordingly, $\alpha = 0.5$ was adopted to balance noise reduction and structural integrity.

The temperature parameter $\tau $ in the softmax function was then evaluated (Fig. 6c). The best performance was consistently achieved at $\tau =0.1$. Smaller values produced overly sharp distributions and unstable gradients, whereas larger values led to over-smoothing of the similarity differences between cells and prototypes, which in turn reduced the model’s discriminative ability. A moderate $\tau $ therefore provided stable optimization while maintaining discriminative capacity.

Finally, we assessed the prototype separation threshold $\beta $ (Fig. 6d). Performance peaked at $\beta = 0.5$. Smaller values imposed weak separation constraints, whereas larger values hindered optimization by making the constraint difficult to satisfy. A moderate $\beta $ balanced inter-type separation and training stability. Together, these findings indicate that PromptSTG maintained stable performance across reasonable parameter ranges, with robustness improving as the number of labeled samples increased.

To systematically evaluate the contribution of each core component to the overall model performance, we conducted an ablation study under a unified few-shot setting. For computational tractability and reproducibility, we derived a standardized subset from hypothalamus sections based on Animal-ID, ensuring that the subset effectively preserved the cell-type distribution and biological complexity of the full dataset. Experimental results (Table 1) demonstrate that the full PromptSTG model—which integrates prompt learning, graph fusion, prototype learning, and prototype separation loss—achieves the highest accuracy score and superior robustness across most settings. Specifically, the necessity of prompt learning is underscored by an $\sim $5% decline in accuracy upon its removal, suggesting that prompt vectors not only facilitate the establishment of fundamental cell type boundaries during pre-training but also enhance adaptability to downstream tasks by refining cellular representations. Furthermore, the exclusion of prototype learning leads to a significant performance decay, validating the critical role of the few-shot learning paradigm in maintaining generalization under extremely limited supervision. Analysis of the graph fusion mechanism reveals that accuracy drops sharply when relying solely on the spatial subgraph, emphasizing that transcriptional similarity is indispensable for resolving cellular heterogeneity; conversely, the performance deficit observed when using only the cell expression subgraph suggests that spatial constraints are essential for capturing the underlying tissue topology. Finally, while the prototype separation loss yields modest gains in raw accuracy, it effectively increases the distances between distinct categories in the embedding space, thereby constructing clearer decision boundaries and bolstering the biological interpretability of the model. Collectively, these findings indicate that the performance advantages of PromptSTG stem from the synergistic gain of its integrated modules rather than the simple addition of individual components.

**Table 1:** Ablation study of PromptSTG components in terms of accuracy (mean $\pm $ std ).

Experiment	5-shot	10-shot	20-shot
w/o Prompt learning	0.647 $\pm $ 0.086	0.696 $\pm $ 0.056	0.733 $\pm $ 0.045
w/o Cell expression subgraph	0.197 $\pm $ 0.043	0.230 $\pm $ 0.042	0.254 $\pm $ 0.045
w/o Spatial information subgraph	0.657 $\pm $ 0.082	0.691 $\pm $ 0.077	0.722 $\pm $ 0.058
w/o Prototype learning	0.479 $\pm $ 0.106	0.459 $\pm $ 0.085	0.440 $\pm $ 0.103
w/o Separation & Compact loss	0.695 $\pm $ 0.068	0.751 $\pm $ 0.052	**0.774 $\pm $ 0.045**
**PromptSTG**	**0.697 $\pm $ 0.067**	**0.752 $\pm $ 0.051**	0.774 $\pm $ 0.043

Bold values indicate the best performance among all compared methods.

### Robustness boundary under extreme label scarcity

To further investigate the robustness boundary of the model under extremely label-scarce conditions, we conducted a challenging gradient down-sampling experiment on the mouse brain dataset. Specifically, we progressively reduced the labeling budget from a 5-shot setting to a 1-shot setting (i.e. only a single labeled sample per class).

**Figure 7.**
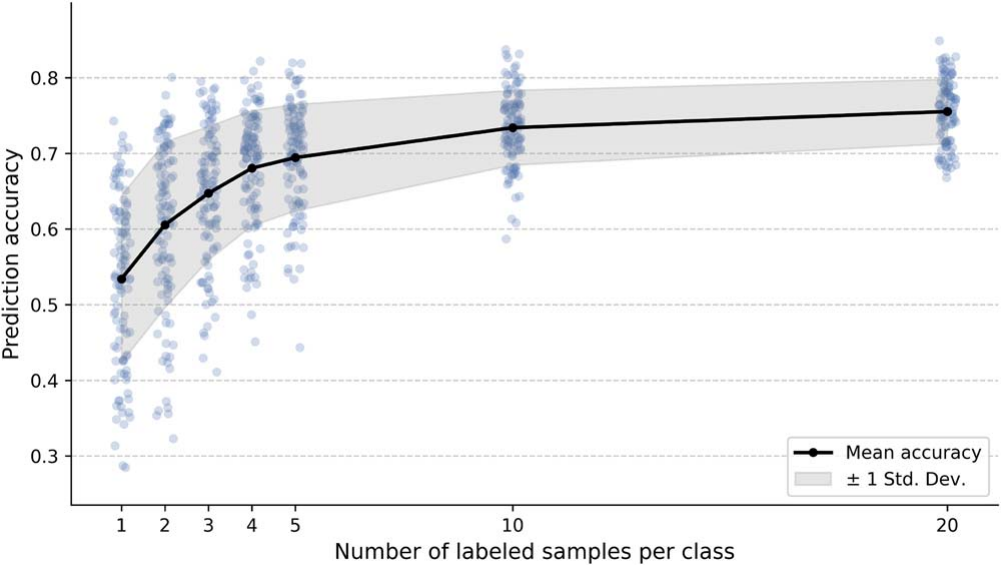
Robustness analysis of PromptSTG performance across few-shot settings ranging from 1-shot to 20-shot.

We observed that under the extreme 1-shot setting, the model still achieves an average accuracy of 0.534; however, its performance exhibits substantial fluctuations, with the minimum accuracy dropping to 0.285 (Fig. 7). This quantitatively demonstrates the instability induced by extreme data scarcity on the lower bound of model performance. Notably, as the number of labeled samples increases, this variability rapidly diminishes. When the number of labeled samples per class reaches $\sim $four (4-shot), the model enters a stable regime, where the minimum accuracy consistently remains around 0.5.

## Discussion and conclusion

We present PromptSTG, a prompt-based framework that enables accurate few-shot cell type annotation across multiple ST platforms (MERFISH, Stereo-seq, and Xenium). The method consistently outperforms existing approaches across datasets and demonstrates particularly strong performance in identifying rare cell types when labeled samples are limited.

The advantage of PromptSTG lies in its ability to overcome key limitations of current methods. Conventional approaches are often sensitive to technical noise and perform poorly when distinguishing rare or transcriptionally similar cell types. In contrast, PromptSTG leverages prototype-guided prompting to achieve stable and reliable annotation even under extremely limited supervision. Moreover, by jointly modeling spatial information and cell expression, PromptSTG captures complex tissue relationships more accurately than methods based solely on spatial or feature information. Compared with foundation models designed for ST, PromptSTG does not require large-scale pretraining yet still exhibits clear advantages in fine-grained, tissue-level classification. For example, on the mouse hypothalamus dataset, the maximum accuracy of SPELL [41] in a zero-shot setting was only 0.640 and it failed to reliably distinguish rare populations, whereas PromptSTG achieved robust and precise annotations even under lower few-shot settings. These results indicate that PromptSTG achieves an effective balance between data efficiency and classification accuracy in ST.

Despite these strengths, PromptSTG has several limitations that point to future directions. First, its performance depends on the quality of the few labeled cells and may be affected by measurement noise or batch effects. Second, the framework currently relies solely on transcriptomic data; integrating histology images or multi-omic information would provide deeper biological insight. Finally, broader validation across multi-center cohorts and diverse platforms is necessary to assess its robustness in real-world applications.

**Key Points**

- PromptSTG enables accurate cell type annotation in spatial transcriptomics under extremely sparse supervision.
- The framework effectively propagates limited labeled information to large unlabeled cell populations by integrating spatial context and cell expression patterns.
- PromptSTG demonstrates robust and scalable performance across diverse tissues, platforms, and challenging few-shot annotation settings.

## Supplementary Material

Supplementary_Information_bhag114

supply-7-5_bbag401

brain_prediction_detail_bbag401

## Data Availability

All datasets used in this paper are publicly available: (1) MERFISH mouse hypothalamus dataset https://datadryad.org/dataset/doi:10.5061/dryad.8t8s248; (2) Stereo-seq COAD dataset https://spatch.pku-genomics.org/#/dataset/stereo; (3) Xenium human breast cancer dataset https://www.10xgenomics.com/products/xenium-in-situ/preview-dataset-human-breast.
