# Supplementary material for: PromptSTG: prototype-guided prompting for few-shot spatial transcriptomics annotation
Source: Brief Bioinform. 2026 Jul 29;27(4):bbag401. doi: 10.1093/bib/bbag401 (PMC13418865; doi:10.1093/bib/bbag401)
Supplement: Supplementary_Information_bhag114 [file supplementary_information_bhag114.docx]

**Supplementary Material**

**Aging shifts hippocampal reactivation from selective reinstatement to category-level misbinding during episodic memory**

Destaw Bayabil Mekbib ^1^, ORCID 0009-0000-2598-9036

Ian M. McDonough*^1,2^, ORCID 0000-0003-0907-8931

^1^ Department of Psychology, Binghamton University, Binghamton, NY 13902, USA

^2^ Center for Cognitive Applications, Binghamton University, Binghamton, NY 13902, USA

Corresponding Author:

Ian M. McDonough, Department of Psychology, Center for Cognitive Applications, Binghamton University, 4400 Vestal Parkway East, Binghamton, NY 13902 USA. Email: [imcdonough@binghamton.edu](mailto:imcdonough@binghamton.edu) Phone: 607-777-4374.

**SI INTRODUCTION**

**Testing Encoding and Attention Accounts**

Some memory theories suggest that the errors exhibited in older adults derive from deficits in attention, thereby impacting memory formation (Naveh-Benjamin 2000; Campbell et al. 2010). If poor binding occurred at encoding as suggested by the Associative Deficit Hypothesis, then older adults would be less likely to reactivate the neural signals at retrieval, potentially explaining the deficits in total accuracy with age and lower encoding-retrieval (EN-RE) overlap in older adults. If older adults had misbinding, as suggested by the hyperbinding account, then reactivation might lead to systematic errors is associative memory similar to those found behaviorally in the study and the relationship between reactivation and different-category errors. We tested these possibilities through the same procedures mentioned earlier but using mean ReHo during encoding a) to predict hippocampal RSI (controlling for age and age interactions) and b) to account for hippocampal connectivity at encoding to and re-tested the hippocampal RSI relationships with behavior. Lastly, we also tested whether measures of attentional control could explain the relationships with behavior.

**SI MATERIALS AND METHODS**

**Hippocampus ROI Definition**

High-resolution T1-weighted structural images were used to define the hippocampus as the primary region of interest (ROI). The hippocampus was segmented using FreeSurfer v6.0 (<http://surfer.nmr.mgh.harvard.edu>). After segmentation, the left and right hippocampal masks were concatenated to form bilateral hippocampal ROI. BOLD signals were subsequently extracted from the concatenated ROIs for all main analyses. For volumetric analyses, the hippocampal volumes were corrected for intracranial volume using the estimated total intracranial volume estimated in Freesurfer using recommended methods (Buckner et al., 2004.)

**Neuropsychological Battery**

***Attention Network Test (ANT).*** This test was developed by (Fan et al. 2002) to test attention and has been shown to reliably differ as a function of age (Ian M McDonough et al. 2019). For this study, alerting and orienting were chosen as the tests for attention. Participants spent 30 minutes determining whether the center arrow points left or right. Sometimes before a trial, a cue in the form of an asterisk above or below a central fixation point alerts participants of an upcoming arrow (single cue), whereas other times a cue appears both above and below a central fixation point (double cue), and yet other times no cue appears (no cue). When the arrow appears, it can be above or below the focal point and can be accompanied by flankers (distracting arrows) that are in the same direction as the center arrow (congruent) or a different direction as the center arrow (incongruent). Alerting is calculated by subtracting RTs in the double-cue condition (i.e., pre-arrow warning) from the no-cue condition. Orienting is calculated by subtracting RTs in the single spatial cue condition (i.e., cue above or below the center that indicates valid information as to where the arrow might appear) from the no cue condition.

**Motion-based volume censoring**

Additional preprocessing steps were applied to the functional data to reduce motion-related and physiological noise. First, volume censoring was performed across all functional runs using a framewise displacement (FD) threshold. FD was calculated from the six rigid-body motion parameters estimated during the motion correction step. Any volume with FD exceeding 0.5 mm was flagged for removal, along with its immediately preceding and following volumes (n−1, n, n+1), following previous methods (King et al. 2022). Participants were excluded from further analysis if more than 50% of volumes in any individual run (R1, EN1, EN2, R2, RE1, or RE2) exceeded this threshold, in line with established practices for identifying motion-contaminated data (Mekbib et al. 2024). The mean percentages of excluded volumes per run were as follows: R1 = 18.11 ± 15.34%, EN1 = 20.18 ± 15.94%, EN2 = 15.74 ± 14.58%, R2 = 24.60 ± 18.44%, RE1 = 18.72 ± 14.68%, and RE2 = 15.85 ± 15.78% (Supplementary Table 2). As expected, motion-related data loss was greater in middle-aged and older adults relative to younger adults, although the overall proportion of censored volumes remained moderate and well below the exclusion threshold. Following volume censoring, nuisance regression was performed on the remaining time series within predefined regions of interest (ROIs) to further remove physiological and motion-related noise. Nuisance regressors included the first five principal components extracted from WM and CSF signals using masks derived from structural segmentation, along with the six rigid-body motion parameters (Tambini and Davachi 2013; King et al. 2022). Finally, the cleaned time series from the encoding (EN1 and EN2) and retrieval (RE1 and RE2) runs were concatenated to create continuous datasets for each task phase.

**Proportion of Variance Explained for the Memory Outcomes**

For each of the memory outcomes, we conducted hierarchical regression to assess the contribution of each set of brain and attention metrics (Supplemental Table 3). The first block was age only, followed by the three RSI metrics (EN-RE, EN-R2, R2-RE), mean ReHo (Baseline and R2), hippocampal volume (left and right), attention (alerting and orientation), and lastly all interactions with age. The proportion of age-related variance accounted for was calculated by the equation:

(b_age_only_ – b_age_and_measures_) / b_age_only_

Where b represents the age beta-coefficient for the age only model (Model 1) or the model with all main effects (Model 5).

**SI RESULTS**

**Hippocampal–Cortical Reactivation Coordination Across Memory Phases**

To determine whether hippocampal reactivation is coordinated with distributed cortical memory representations, we examined hippocampal–cortical reactivation coupling across EN–R2, EN–RE, and R2–RE phases. Cortical regions included the parahippocampal cortex (PRHC), dorsomedial prefrontal cortex (DMPFC), precuneus (PreCu), medial temporal gyrus (MTG), lateral occipital cortex (LOC), fusiform gyrus (FFG), insula, entorhinal cortex (Ento), and amygdala (Amy). ROI-specific linear models tested age-dependent hippocampal–cortical coordination while controlling for sex, education, and regional homogeneity (ReHo).

As shown in Figure S2, age-dependent hippocampal–cortical reactivation coordination varied across cortical regions and memory phases. The strongest effect was observed for HC–LOC during Encoding–Rest (β = 0.82, FDR-corrected p < .05), indicating robust age-related modulation of hippocampal–visual association coordination during post-encoding consolidation. Additional effects were observed for HC–PreCu during Encoding–Retrieval (β = 0.55, p < .05, uncorrected) and for HC–Insula during Encoding–Rest (β = 0.59), Encoding–Retrieval (β = 0.60), and Rest–Retrieval (β = 0.68). In contrast, PRHC, MTG, FFG, Ento, Amy, and DMPFC showed weaker and non-significant effects. Overall, these findings suggest that age-related differences in hippocampal–cortical reactivation are selectively expressed within visual association, default-mode, and salience-related systems across learning, consolidation, and retrieval phases.

**Hippocampal–Cortical Reactivation Coupling as a Predictor of Memory Performance**

We next examined whether hippocampal–cortical reactivation coupling predicted memory performance. Linear regression models tested the effects of HC–cortical coupling, age, and their interaction on Total Accuracy, Forgetting, and Category Error Bias while controlling for sex, education, and regional homogeneity. No main effects of hippocampal–cortical coupling on memory performance survived multiple-comparison correction (all FDR-corrected p > .05). In contrast, significant HC–cortical coupling × age interactions were observed for Total Accuracy and HC–Insula coupling during Encoding–Retrieval (β = -0.218, t = -2.31, p = .025), Forgetting and HC–Insula coupling during Encoding–Retrieval (β = 0.404, t = 2.95, p = .005), Category Error Bias and HC–PreCu coupling during Encoding–Retrieval (β = -0.385, t = -3.06, p = .003), and Category Error Bias and HC–Insula coupling during Encoding–Rest (β = -0.430, t = -2.77, p = .008), Encoding–Retrieval (β = -0.292, t = -2.76, p = .008), and Rest–Retrieval (β = -0.497, t = -2.88, p = .006). These findings indicate that the relationship between hippocampal–cortical reactivation coordination and memory performance differ as a function of age.

As illustrated in Figure S3, the association between hippocampal–cortical reactivation coupling and memory performance differed substantially across the adult lifespan. For Total Accuracy, stronger HC–Insula coupling during Encoding–Retrieval was associated with higher memory accuracy in younger adults (β = 0.514, p = .006), but this relationship progressively weakened with age and was absent in older adults (β = -0.008, p = .950). A similar pattern was observed for Forgetting, where stronger HC–Insula coupling during Encoding–Retrieval was associated with reduced forgetting in younger adults (β = -0.871, p = .001), whereas this relationship was not evident in middle-aged or older adults. A distinct pattern emerged for Category Error Bias. In older adults, stronger HC–PreCu and HC–Insula coupling was consistently associated with fewer same-category false alarms across multiple memory phases. This relationship was most pronounced for HC–PreCu coupling during Encoding–Retrieval (β = -0.425, p = .006) and HC–Insula coupling during Rest–Retrieval (β = -0.606, p = .003). In contrast, younger adults exhibited positive or near-zero associations between hippocampal–cortical coupling and Category Error Bias, resulting in an age-related reversal of the coupling–behavior relationship.

**Attentional Processes and Relationships with RSI**

We examined whether attentional processes could explain these effects. Our two attentional control measures were not correlated with age (rs < .12, ps > .38). Neither alerting (Fig. S7) nor orienting (Fig. S8) significantly predicted RSI across memory phases (β ≤ 0.09, p ≥ 0.36). When controlling for alerting, EN–RE RSI continued to predict total accuracy (β = 0.023, p = 0.036), whereas the age and RSI interaction was not significant (β = -0.02, p = 0.07). Age and RSI interactions remained significantly predicted for category error bias (EN–R2: β = -0.038, p = 0.005; EN–RE: β = -0.028, p = 0.014; R2–RE: β = –0.036, p = 0.005) and forgetting (EN–RE: β = 0.04, p = 0.009). Higher RSI was associated with increased different-category error bias in older adults across phases (β<=-0.04, p<=0.01) and reduced forgetting in young adults (β = -0.06, p = 0.01).

A nearly identical pattern emerged when controlling orienting. EN–RE RSI again predicted total accuracy (β = 0.022, p = 0.0476), with no significant age and RSI interaction (β = -0.021, p = 0.067). The age and RSI interactions for category error bias and forgetting remained consistent with the alerting model (category error bias: EN–R2: β = -0.038, p = 0.004; EN–RE: β = -0.03, p = 0.008; R2–RE: β = –0.034, p = 0.008;forgetting:β = 0.042, p = 0.006). As when controlling for alerting, higher RSI was associated with increased different category error bias in older adults across phases (β<=-0.04, p<=0.01) and reduced forgetting errors in young adults (β = -0.07, p < 0.0001).

Table S1. Demographic information as a function of age group

| Factor | Young (20-30) | Middle Age (51-60) | Old (61-74) |
| --- | --- | --- | --- |
| N | 17 | 21 | 23 |
| Age (M/SD) | 23.53/3.20 | 54.67/2.85 | 66.14/4.18 |
| Sex (F/M) | 9/8 | 14/7 | 12/10 |
| Race |  |  |  |
| Non-Hispanic White (%) | 64.7 | 52.38 | 82.61 |
| African American (%) | 0 | 38.09 | 17.39 |
| Other (%) | 35.3 | 9.53 | 0 |
| Education (M/SD) | 15.06/2.14 | 14.67/2.39 | 14.82/2.11 |
| SLUMS (M/SD) | N/A | 27.05/2.69 | 26.48/2.80 |
| Total Accuracy (M [SD]) | 0.36 [0.02] | 0.24 [0.08] | 0.20 [0.06] |
| Forgetting (M [S]) | 0.16 [0.13] | 0.17 [0.09] | 0.15 0.11] |
| Category error bias (M [SD]) | 0.22 [0.01] | 0.22 [0.08] | 0.27 [0.07] |

Table S2. Summary of excluded volumes.

| Group | Rest 1  Mean [SD] | Encoding Mean [SD] | Rest 2  Mean [SD] | Retrieval Mean [SD] | Overall  Mean [SD] |
| --- | --- | --- | --- | --- | --- |
| Young | 8.22 [9.92] | 10.89 [10.87] | 17.78 [13.99] | 10. 97[14.80] | 11.96 [10.11] |
| Middle age | 21.03 [22.35] | 27.29 [24.61] | 34.07 [24.94] | 29.24 [25.28] | 27.91 [21.31] |
| Old | 24.87 [20.66] | 31.25 [19.59] | 43.40 [22.92] | 33.71 [25.36] | 33.31 [18.45] |

Table S3. Proportion of Variance Explained for Memory Outcomes

| **Outcome** | **Model** | **Added Predictors** | **R^2^** | **F** | **df** | **Sig** | Δ **R^2^** | Δ **F** | Δ **Sig** |
| --- | --- | --- | --- | --- | --- | --- | --- | --- | --- |

| Total Accuracy^a^ | 1 | Age | 0.39 | 37.78 | 59 | <.001 | - | - | - |
| --- | --- | --- | --- | --- | --- | --- | --- | --- | --- |
|  | 2 | RSI | 0.45 | 11.25 | 56 | <.001 | 0.06 | 1.88 | 0.15 |
|  | 3 | mReHo | 0.47 | 7.87 | 54 | <.001 | 0.02 | 1.06 | 0.35 |
|  | 4 | Volume | 0.48 | 6.05 | 52 | <.001 | 0.01 | 0.81 | 0.45 |
|  | 5 | Attention | 0.53 | 5.70 | 50 | <.001 | 0.05 | 2.58 | 0.09 |
|  | 6 | Interactions | 0.60 | 3.21 | 41 | <.001 | 0.07 | 0.74 | 0.67 |
| Forgetting^b^ | 1 | Age | 0.00 | 0.05 | 59 | 0.83 | - | - | - |
|  | 2 | RSI | 0.04 | 0.51 | 56 | 0.73 | 0.04 | 0.74 | 0.54 |
|  | 3 | mReHo | 0.04 | 0.33 | 54 | 0.92 | 0.00 | 0.01 | 0.99 |
|  | 4 | Volume | 0.12 | 0.87 | 52 | 0.55 | 0.08 | 2.65 | 0.08 |
|  | 5 | Attention | 0.12 | 0.69 | 50 | 0.73 | 0.00 | 0.13 | 0.88 |
|  | 6 | Interactions | 0.37 | 1.23 | 41 | 0.28 | 0.25 | 1.73 | 0.11 |
| Category Bias Error^c^ | 1 | Age | 0.03 | 2.02 | 59 | 0.16 | - | - | - |
|  | 2 | RSI | 0.08 | 1.23 | 56 | 0.31 | 0.05 | 1.23 | 0.31 |
|  | 3 | mReHo | 0.16 | 1.76 | 54 | 0.12 | 0.08 | 3.23 | 0.05 |
|  | 4 | Volume | 0.16 | 1.28 | 52 | 0.28 | 0.00 | 0.01 | 0.99 |
|  | 5 | Attention | 0.19 | 1.18 | 50 | 0.32 | 0.03 | 1.07 | 0.35 |
|  | 6 | Interactions | 0.47 | 1.93 | 41 | 0.04 | 0.28 | 2.42 | 0.03 |
| a = Brain and attention measures collectively account for 31.53% of the age-related variance in total accuracy. | | | | | | | | | |

| b = Age-related differences in memory performance are largely accounted for by brain measures, leaving little independent effect of chronological age. |
| --- |

| c = Brain and attention measures collectively account for 60.32% of the age-related variance in category error bias. |
| --- |

**
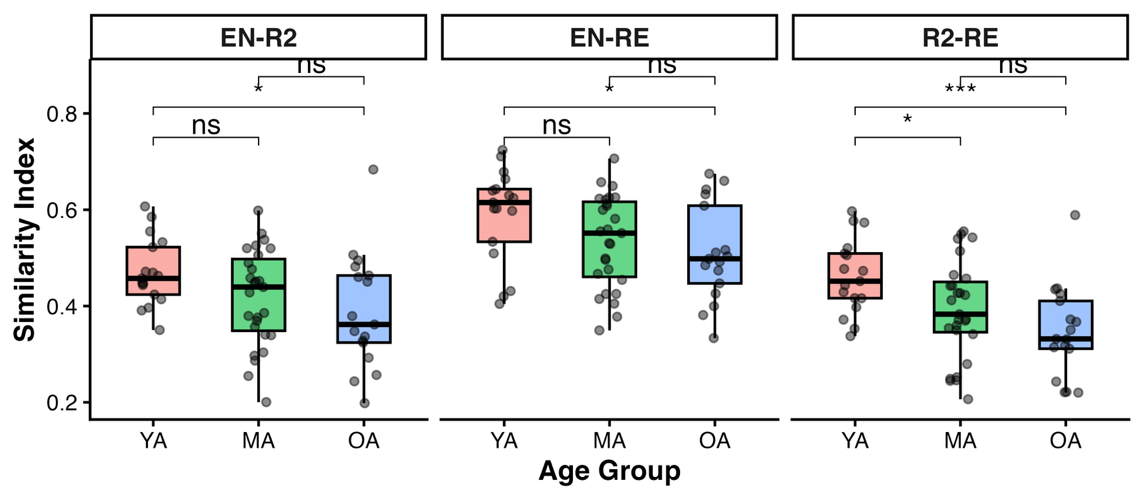
**

**Figure S1.** Hippocampal representational similarity index (RSI) across categorical age groups and memory phases. Boxplots show hippocampal RSI for EN–R2, EN–RE, and R2–RE memory phases across young adults (YA; 20–30 years), middle-aged adults (MA; 50–63 years), and older adults (OA; 63–74 years). Individual data points are overlaid. Consistent with the continuous-age analyses, YA generally exhibited higher RSI values than OA across memory phases, whereas differences between MA and OA were less consistent. Pairwise group comparisons are indicated above each panel (*p < .05, ***p < .001, ns = not significant). These categorical analyses were conducted to further evaluate age-related effects given the discontinuity in the sampled age range between approximately 30–50 years.


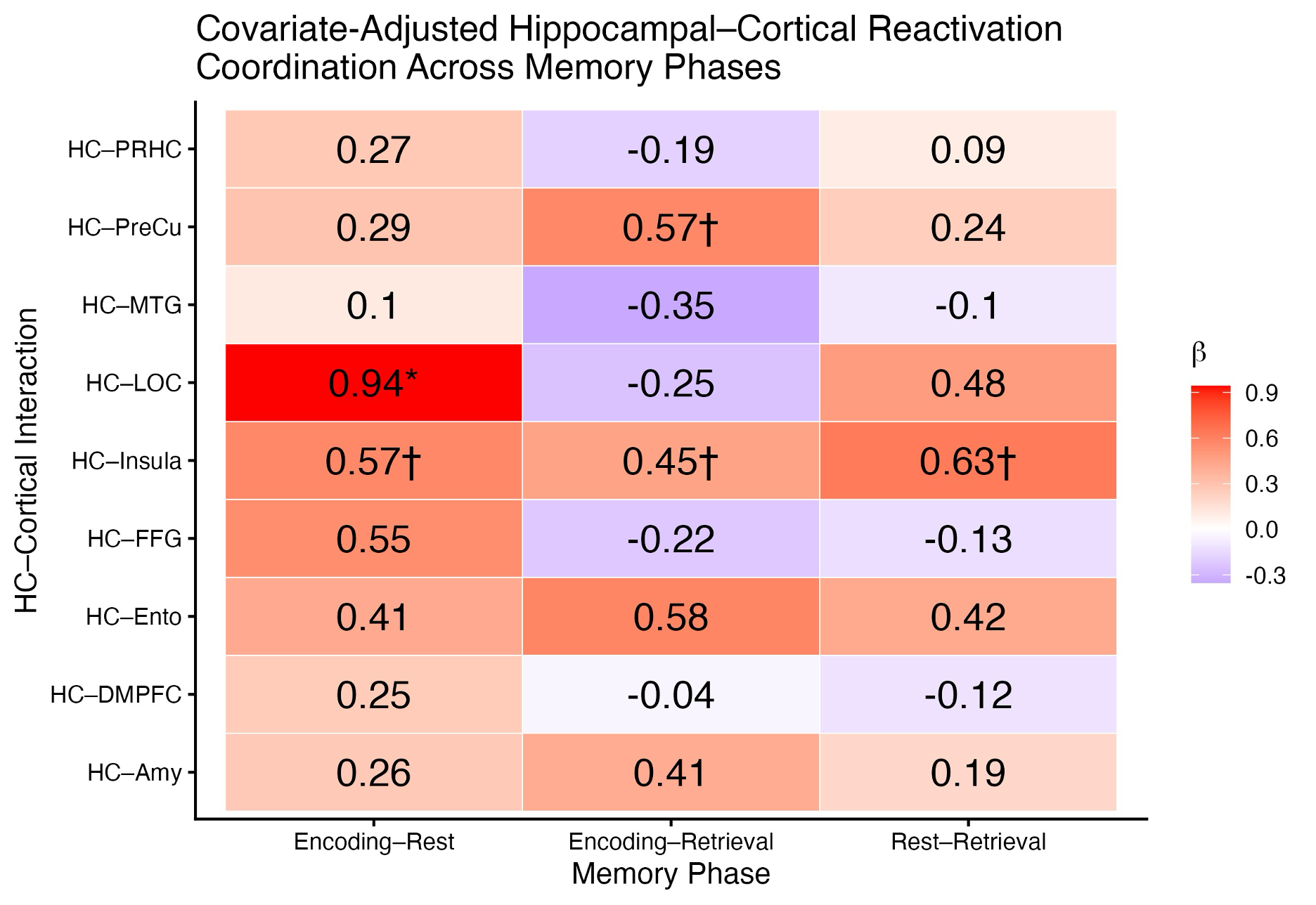


**Figure S2.** Age-dependent hippocampal–cortical reactivation coordination across memory phases. Heatmap showing age-dependent hippocampal–cortical reactivation coordination across Encoding–Rest, Encoding–Retrieval, and Rest–Retrieval phases. For each cortical ROI, linear regression models assessed the HC × Age Group interaction while adjusting for sex, education, and regional homogeneity (ReHo). Cell values represent the β coefficients of the interaction term. Significant effects are indicated by symbols (* FDR-corrected p < .05; † uncorrected p < .05).


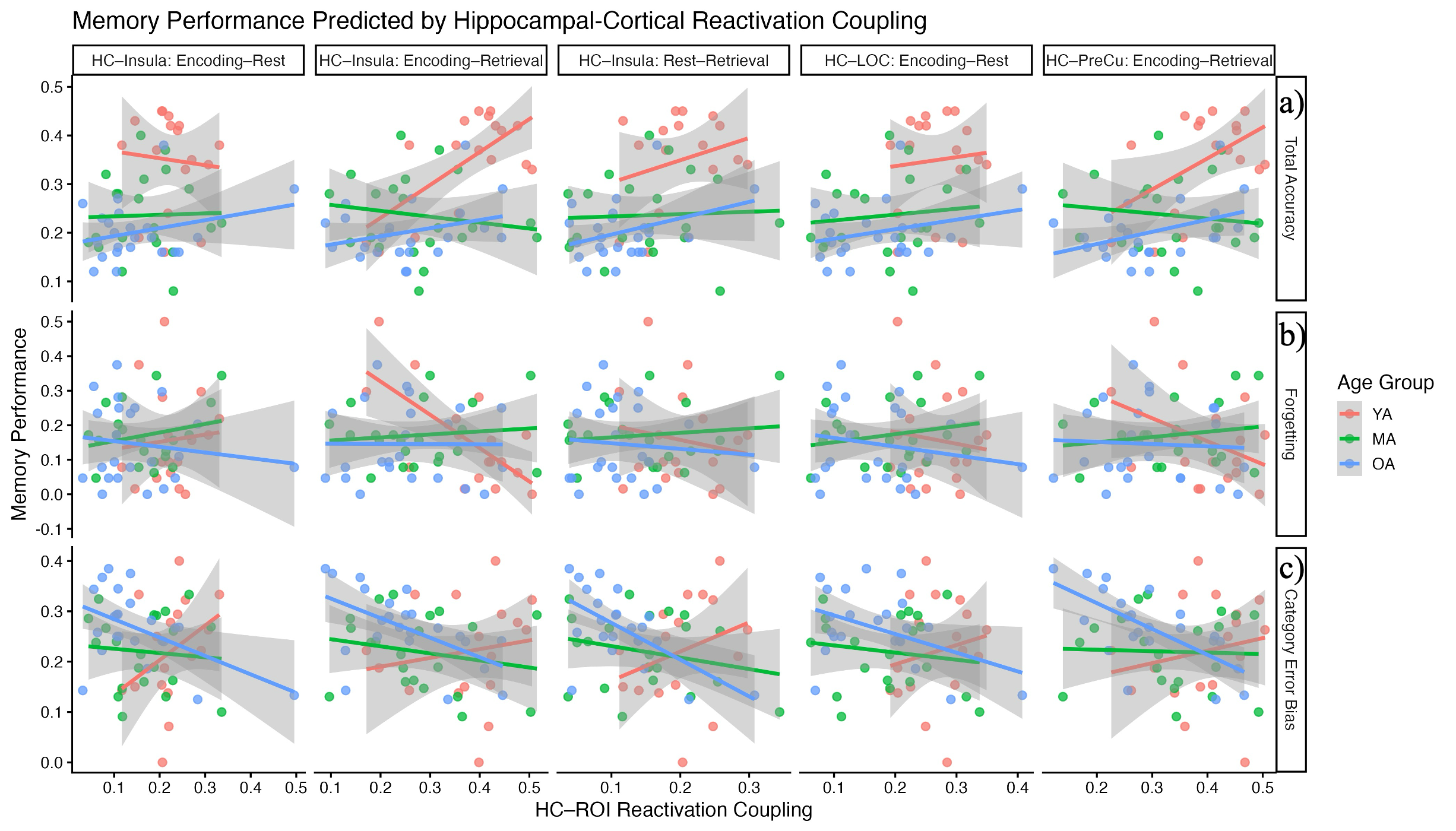


**Figure S3.** Memory performance predicted by hippocampal–cortical reactivation coupling. Scatterplots illustrate significant hippocampal–cortical (HC–cortical) pathways identified from the HC–cortical Coupling × Age interaction analyses. Panels show associations between HC–cortical reactivation coupling and (a) Total Accuracy, (b) Forgetting, and (c) Category Error Bias across memory phases. Regression lines represent simple slopes estimated at representative younger adult (YA), middle-aged adult (MA), and older adult (OA) ages, with shaded regions indicating 95% confidence intervals. Significant interactions indicate that the relationship between hippocampal–cortical reactivation coordination and memory performance differs across the adult lifespan.


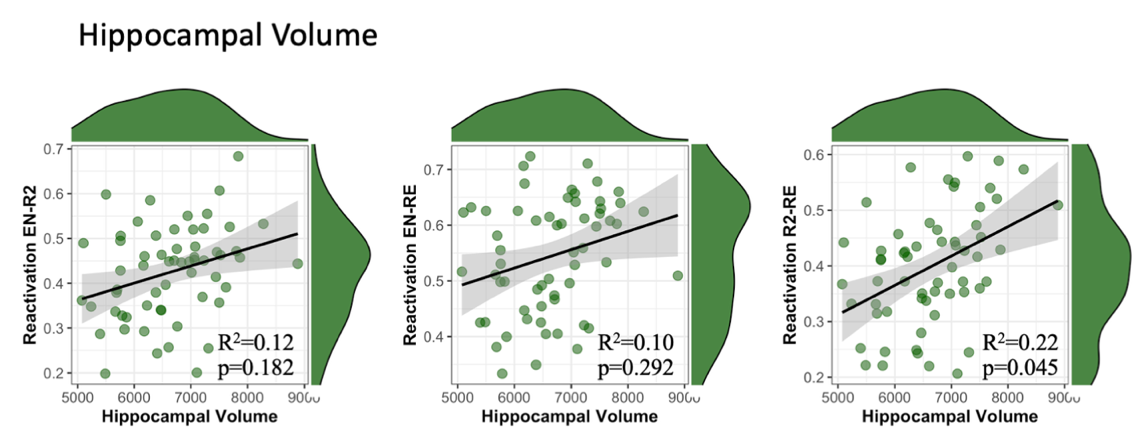


**Figure S4.** Relationship between hippocampal volume and multivoxel pattern similarity across memory phases. We tested whether individual differences in hippocampal volume predicted the degree of hippocampal representational similarity (RSI) across encoding, post-encoding rest, and retrieval. Volume significantly predicted R2–RE RSI but not EN–R2 or EN–RE RSI, indicating that hippocampal recapitulation is largely independent of overall hippocampal size across the adult lifespan. Abbreviations: EN–R2 = encoding–post-encoding rest similarity, EN–RE = encoding–retrieval similarity, R2–RE = post-encoding rest–retrieval similarity, RSI = representational similarity index.


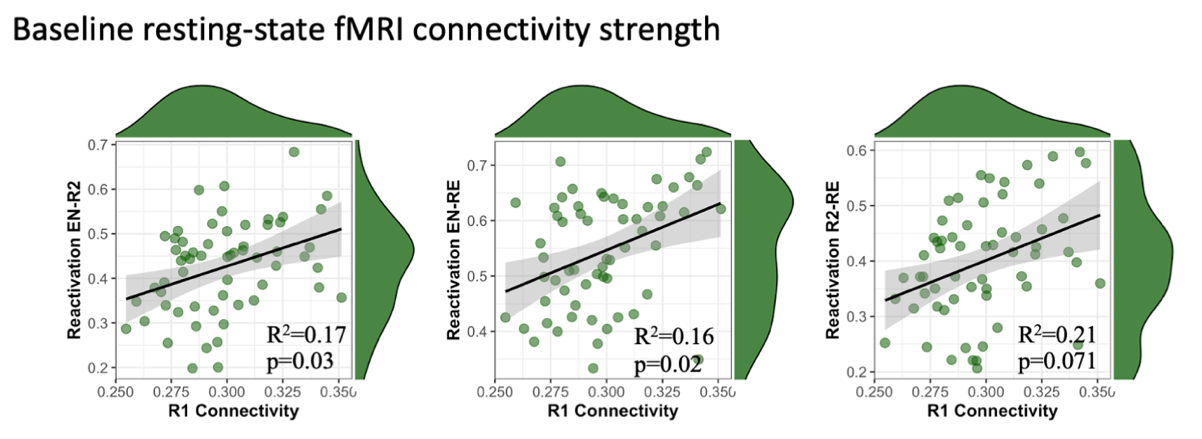


**Figure S5.** Baseline hippocampal connectivity strength and subsequent multivoxel pattern similarity. Baseline hippocampal mReHo, measured during the pre-encoding rest scan, predicted EN–R2 and EN–RE representational similarity indices but not R2–RE RSI. No significant interactions with age were observed, indicating that these effects were consistent across the adult lifespan. Abbreviations: EN–R2 = encoding–post-encoding rest similarity, EN–RE = encoding–retrieval similarity, R2–RE = post-encoding rest–retrieval similarity, RSI = representational similarity indices, mReHo = mean regional homogeneity.


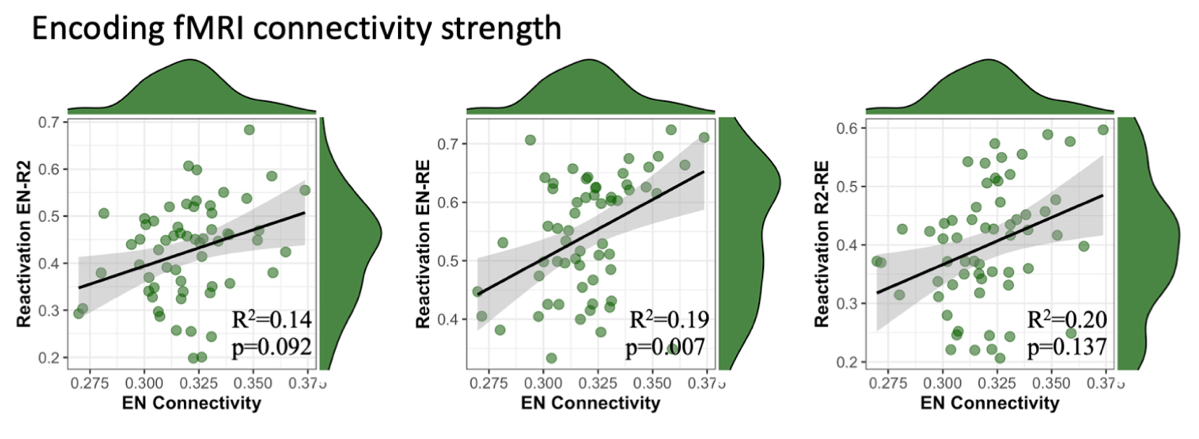


**Figure S6.** Encoding hippocampal connectivity and subsequent multivoxel pattern similarity. Hippocampal mReHo during encoding predicted EN–RE RSI, indicating that stronger hippocampal connectivity at encoding facilitates direct encoding-to-retrieval recapitulation. Encoding mReHo did not significantly predict EN–R2 or R2–RE RSI, nor did it interact with age across memory phases. Abbreviations: EN–R2 = encoding–post-encoding rest similarity, EN–RE = encoding–retrieval similarity, R2–RE = post-encoding rest–retrieval similarity, RSI = representational similarity indices, mReHo = mean regional homogeneity


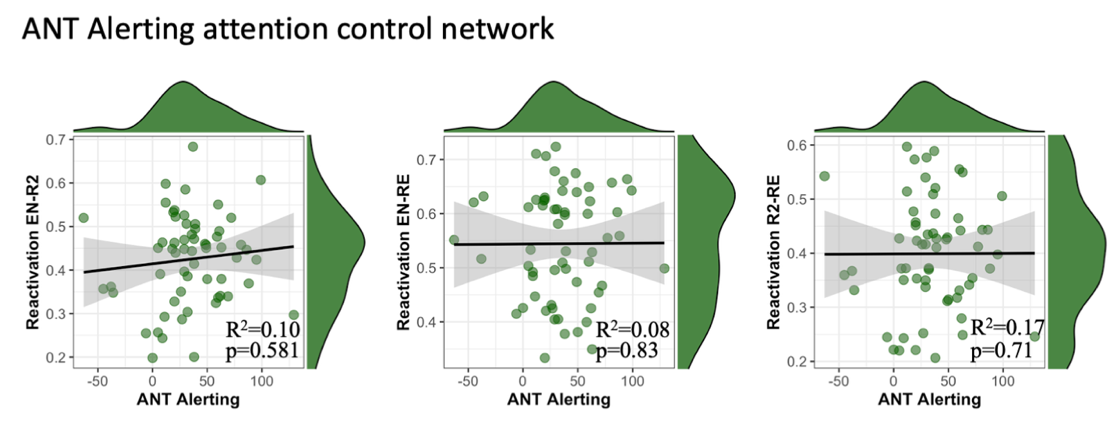


**Figure S7.** ANT alerting and subsequent multivoxel pattern similarity. ANT alerting did not significantly predict RSI nor did it interact with age across memory phases. Abbreviations: ANT = Attention Network Test, EN–R2 = encoding–post-encoding rest similarity, EN–RE = encoding–retrieval similarity, R2–RE = post-encoding rest–retrieval similarity, RSI = representational similarity indices.


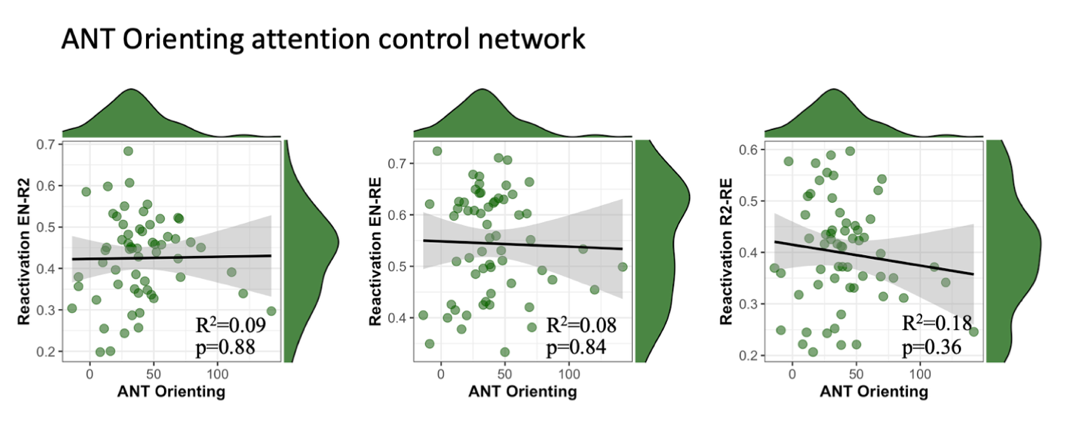


**Figure S8.** ANT orienting and subsequent multivoxel pattern similarity. ANT orienting did not significantly predict RSI nor did it interact with age across memory phases. Abbreviations: ANT = Attention Network Test, EN–R2 = encoding–post-encoding rest similarity, EN–RE = encoding–retrieval similarity, R2–RE = post-encoding rest–retrieval similarity, RSI = representational similarity indices.

**References**

Buckner RL et al. 2004. A unified approach for morphometric and functional data analysis in young, old, and demented adults using automated atlas-based head size normalization: reliability and validation against manual measurement of total intracranial volume. Neuroimage. 23(2):724-38. https://doi: 10.1016/j.neuroimage.2004.06.018.

Campbell KL, Davis EE. 2024. Hyper-Binding: Older Adults Form Too Many Associations, Not Too Few. Curr Dir Psychol Sci. 33(5):292–299. https://doi.org/10.1177/09637214241263020

Fan J et al. 2002. Testing the Efficiency and Independence of Attentional Networks. J Cogn Neurosci. 14(3):340–347. https://doi.org/10.1162/089892902317361886

Ian M McDonough, Meagan M Wood, William S Miller Jr. 2019. A Review on the Trajectory of Attentional Mechanisms in Aging and the Alzheimer’s Disease Continuum through the Attention Network Test. Yale J Biol Med. 92(1):37

King BR et al. 2022. Persistence of hippocampal and striatal multivoxel patterns during awake rest after motor sequence learning. iScience. 25(12). https://doi.org/10.1016/j.isci.2022.105498

Mekbib DB et al. 2024. Reproducibility and Sensitivity of Resting-State fMRI in Patients With Parkinson’s Disease Using Cross Validation-Based Data Censoring. Journal of Magnetic Resonance Imaging. 59(5):1630–1642. <https://doi.org/10.1002/jmri.28958>

Naveh-Benjamin M. 2000. Adult age differences in memory performance: Tests of an associative deficit hypothesis. J Exp Psychol Learn Mem Cogn. 26(5):1170–1187. https://doi.org/10.1037/0278-7393.26.5.1170

Tambini A, Davachi L. 2013. Persistence of hippocampal multivoxel patterns into postencoding rest is related to memory. Proc Natl Acad Sci U S A. 110(48):19591–19596. https://doi.org/10.1073/pnas.1308499110
