# Supplementary material for: PromptSTG: prototype-guided prompting for few-shot spatial transcriptomics annotation
Source: Brief Bioinform. 2026 Jul 29;27(4):bbag401. doi: 10.1093/bib/bbag401 (PMC13418865; doi:10.1093/bib/bbag401)
Supplement: supply-7-5_bbag401 [file supply-7-5_bbag401.pdf]

Supplementary information

PromptSTG: Prototype-Guided Prompting for Few-shot Spatial Transcriptomics Annotation

Table of Contents

Supplementary Text 3

Implementation details . . . 3

Experimental setup . . . 3

Computational efficiency . . . 3

Activation function . . . 4

Graph fusion . . . 5

Evaluation metrics . . . 6

Supplementary Figures 7

Supplementary Tables 15

Summary of cell and gene numbers in the preprocessed datasets . . . 15

Accuracy comparison on 36 mouse hypothalamus sections (MERFISH). . . . 15

Accuracy comparison on the colorectal adenocarcinoma dataset (COAD, Stereo-seq) . . . 15

Accuracy comparison on the human breast cancer dataset (Xenium) . . . 16

Supplementary Text

Implementation details

PromptSTG was implemented using the PyTorch framework. During the graph representation pretraining stage, the Adam optimizer was employed with a learning rate of 0.001 and a weight decay of 0.001. The hidden dimension was set to 16, and ELU was used as the activation function (the choice of activation function will be discussed in the supplementary materials). Early stopping was applied when the loss did not decrease for a certain number of epochs (patience = 20), with a maximum of 10,000 training epochs and a batch size of 1.

In the graph prompting stage, the learning rate was set to 0.01, and the model was trained for a fixed number of epochs. Specifically, 30 epochs were used for the weak-link removal fusion strategy, while 5 epochs were used for the normalized fusion strategy (weak-link removal fusion typically requires more iterations due to structural refinement, whereas normalized fusion converges more rapidly due to its scale alignment nature). Under the few-shot setting, we performed class-balanced sampling by selecting k labeled instances per cell type to construct the training set, while all remaining samples are used for testing (k-shot).

In all experiments, the number of neighbors K for each cell in the gene expression subgraph was set to 200, the threshold α was set to 0.5, the temperature parameter τ was set to 0.1, and the prototype distance threshold β was set to 0.5.

Experimental setup

To compare PromptSTG with seven baseline methods (scmap, Cell2location, Tangram, Spatial-ID, DSCT, scPoli, and jMF2D), we conducted all experiments under three few-shot settings (5, 10, and 20 samples per class), representing scenarios with extremely limited labeled cells. As described in the Evaluation Metrics section, accuracy and F1 score were used to assess model performance. For the baseline methods, labeled cells were used as single-cell reference data, while the remaining unlabeled cells were treated as ST data for annotation. Unless otherwise specified, all methods followed the same preprocessing pipeline and used default hyperparameters to ensure a fair comparison. For Spatial-ID, we followed the authors' recommendations and adopted the default PyTorch-based pretraining procedure with a hidden dimension of 16. For Cell2location, gene expression data were retained as integer counts after cell filtering, and posterior estimates were obtained using 100 Monte Carlo samples.

For all methods, quantitative performance was averaged across multiple random seeds (0–9), with seeds 0–4 specifically utilized for the mouse hypothalamus dataset to ensure statistical robustness. Qualitative results are reported based on the model trained with random seed 0. For visualization, only four baseline methods (scmap, Spatial-ID, DSCT, and scPoli) were presented in the main text, while the cell type annotation results for Cell2location, Tangram, and jMF2D were provided in the Supplementary Figures.

In the main text, parameter sensitivity is evaluated under a validation-based early stopping protocol. Specifically, 10% of samples from each class (excluded from training) are used to construct a validation set, and model selection is performed based on validation performance. Importantly, this early stopping setting is not intended to reflect a deployment or final training strategy, but rather serves as a controlled protocol to estimate the best achievable performance under each parameter configuration on the current dataset. For completeness, parameter sensitivity results under standard full-training without early stopping are provided in Supplementary Fig. S7.

Computational efficiency

All experiments were conducted on an NVIDIA RTX 3090 GPU. We report the computational cost of the proposed method in terms of training time and GPU memory usage. Specifically, the average pretraining time is 133.38 s, while the fine-tuning stage requires 30.65 s. The corresponding GPU memory consumption is 2.78 GB for pretraining and 2.74 GB for fine-tuning.

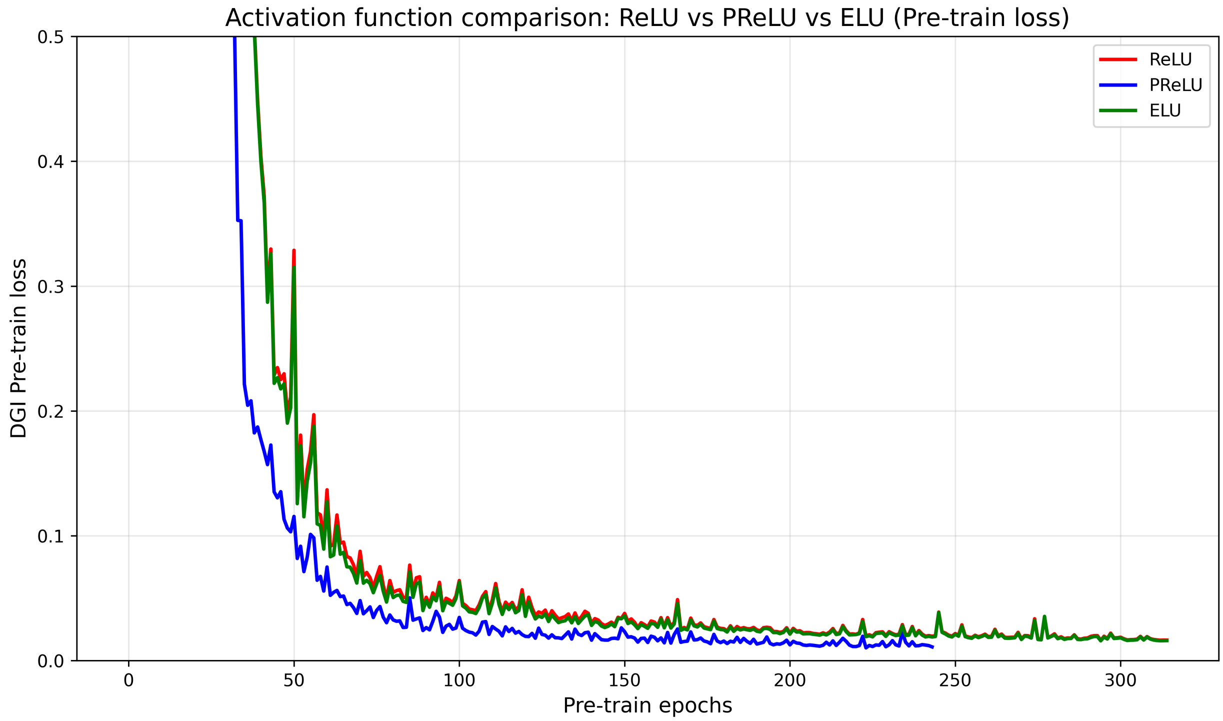

Supplementary Figure 1. Loss convergence of different activation functions during pretraining on the Mouse hypothalamus dataset (Seed 0, 20-shot, mid 1).

Activation function

During model development, we explored three widely used activation functions—ReLU, PReLU, and ELU. We observed differences in optimization efficiency during the pretraining stage, as reflected in their loss convergence behavior. Based on these empirical observations, ELU was selected as the activation function for graph representation pretraining.

From a theoretical perspective, ELU produces non-zero outputs for negative inputs, which helps mitigate the bias shift effect and encourages activations to be more zero-centered. This property may be beneficial in GCNs, where multi-layer neighborhood aggregation can amplify distributional shifts and affect numerical stability. In contrast, while PReLU enhances the modeling capacity for negative inputs through learnable parameters, it also increases model complexity, and this additional flexibility did not yield noticeable benefits in our pretraining setting. ELU, on the other hand, provides a smooth and stable nonlinear transformation without introducing extra parameters.

Supplementary Table 1. Loss convergence comparison of different activation functions during pretraining.

Activation	Epochs	Initial Loss	Final Loss	Total Drop	Avg Drop Speed	Early Speed (first 50)
ReLU	315	160.1372	0.0165	160.1208	0.508320	3.198523
PReLU	244	95.2785	0.0110	95.2675	0.390441	1.903506
ELU	315	160.2487	0.0161	160.2326	0.508675	3.200924

From the perspective of loss convergence, ELU achieves a slightly lower final loss than ReLU (0.0161 vs. 0.0165) and exhibits a marginally faster loss decrease in the early stages of training, indicating more efficient optimization dynamics (Supplementary Fig. S1 and Supplementary Table 1). Although PReLU reaches early stopping in fewer epochs (244 vs. 315), its loss reduction is consistently slower, particularly during the early training phase. This suggests that its additional flexibility does not necessarily translate into faster optimization in terms of convergence behavior.

Overall, ELU offers a favorable balance between optimization dynamics, stability, and model simplicity, making it a suitable choice for the graph representation pretraining stage.

Graph fusion

In this section, we describe the design motivations, biological rationale, and adaptive selection mechanism of the two graph fusion strategies.

For weak-link removal fusion, the primary goal is to identify and eliminate unreliable connections that may cross tissue boundaries. Specifically, we leverage the gene expression graph to filter edges in the spatial graph: if two spatially adjacent nodes exhibit gene expression similarity above a threshold α, the corresponding edge is preserved or strengthened; otherwise, it is weakened or removed. From a biological perspective, this strategy is based on the assumption that cells within the same spatial region are not only physically proximate but also consistent at the molecular level, whereas spatially adjacent cells with substantial expression differences are more likely to belong to distinct functional regions. By introducing a similarity threshold, we explicitly encode regional boundaries into the graph structure, thereby reducing cross-region information propagation and improving the biological fidelity of GCN aggregation.

For normalized fusion, the motivation lies in addressing the inconsistency in weight definitions and scales across different modalities. The spatial graph is typically constructed using Euclidean distance with a Gaussian kernel, while the gene expression graph is based on cosine similarity, leading to substantial differences in value distributions and scales. A direct linear combination of the two adjacency matrices may cause one graph to dominate the aggregation process due to scale mismatch, thereby weakening the complementarity of multimodal information. To mitigate this issue, we adopt a normalized fusion strategy to align the scales of different graphs, ensuring that each modality contributes more evenly during information propagation. From a biological standpoint, this reflects the complementary nature of spatial proximity and molecular similarity in characterizing tissue organization, and emphasizes that both should be jointly modeled rather than dominated by a single modality.

Finally, we adaptively select the fusion strategy based on the spatial–expression consistency of the spatial graph. We characterize the graph using three complementary statistics:

$$H = \frac{\sum_{(i,j)\in E} A_{ij} \cdot (1 - c_{ij})}{\sum_{(i,j)\in E} A_{ij}}, \tag{1}$$

$$s_i = \frac{\sum_{j\in N(i)} A_{ij} \cdot c_{ij}}{\sum_{j\in N(i)} A_{ij}}, \quad \mu = \frac{1}{N}\sum_i s_i, \quad \sigma^2 = \frac{1}{N}\sum_i (s_i - \mu)^2. \tag{2}$$

$$c_{ij} = \frac{x_i x_j^\top}{\|x_i\|_2 \, \|x_j\|_2}, \tag{3}$$

where c_{ij} denotes the cosine similarity between the expression profiles of nodes i and j, H measures the global edge heterogeneity, while s_i captures the local neighborhood consistency of node i. The mean μ and variance σ^2 further describe the distribution of local consistency across the graph.

Based on the above statistical analysis, we design an adaptive fusion strategy according to the structural characteristics of the data. When the spatial graph exhibits local connectivity inconsistency (i.e., high variance of node-level consistency), we apply **weak-link removal fusion** to eliminate unreliable edge noise. In contrast, when the graph structure shows globally low consistency (i.e., both low mean and low variance), it indicates a systematic global decoupling between spatial proximity and gene expression. In this case, we adopt **normalized fusion** to preserve the complementarity between multimodal information.

This design effectively distinguishes between local structural noise and global spatial–expression decoupling, thereby achieving a dynamic balance between structural correction and information integration. In experiments, the MERFISH mouse hypothalamus dataset exhibits pronounced local structural instability, with a global heterogeneity of 0.605, a local consistency mean of 0.395, and a variance of 0.017. After applying weak-link removal fusion, the heterogeneity is significantly reduced to 0.115. The breast cancer Xenium dataset shows similar characteristics, with a relatively high local consistency variance (0.041), indicating local structural inconsistency; after processing, its heterogeneity decreases from 0.564 to 0.157. In

contrast, the COAD dataset presents markedly different statistical properties, with an extremely high global heterogeneity of 0.924, while the local consistency mean (0.076) and variance (0.00065) are both very low. This suggests a system-wide decoupling between spatial adjacency and expression similarity, with almost no localized structural anomalies. Therefore, weak-link removal fusion is no longer appropriate in this setting. Instead, we employ normalized fusion to preserve the complementarity between spatial and expression information, successfully reducing heterogeneity from 0.924 to 0.583.

Evaluation metrics

Accuracy

We used the accuracy score as a metric to evaluate the annotation performance. Test sets contained c cell types. For each cell type i, let a_i and b_i represent the number of correctly and incorrectly assigned cells, respectively.
The accuracy for each cell type is:

$$\text{Accuracy}_c = \frac{a_c}{a_c + b_c},$$

The overall accuracy across all cell types is:

$$\text{Accuracy} = \frac{\sum_{i=1}^{c} a_i}{\sum_{i=1}^{c} (a_i + b_i)},$$

Both a_i and b_i represent values obtained from the test set.

F1-score

We used the F1-score as another metric to evaluate the annotation performance. Test sets contain c cell types, and for each cell type i, TP_i, FP_i, and FN_i represent the number of true positives, false positives and false negatives respectively. The precision and recall are defined as:

$$\text{Precision}_i = \frac{\text{TP}_i}{\text{TP}_i + \text{FP}_i},$$

$$\text{Recall}_i = \frac{\text{TP}_i}{\text{TP}_i + \text{FN}_i}.$$

The F1-score for cell type i is calculated as:

$$\text{F1}_i = \frac{2 \cdot \text{Precision}_i \cdot \text{Recall}_i}{\text{Precision}_i + \text{Recall}_i}.$$

CV

The *coefficient of variation* (CV) is a standardized measure of dispersion that quantifies the relative variability of a distribution. It is defined as the ratio of the standard deviation σ to the mean μ:

$$\text{CV} = \frac{\sigma}{\mu}.$$

A lower CV indicates more stable or homogeneous measurements, whereas a higher CV reflects greater relative variability. When reporting t-CV or the CV at a threshold t, the coefficient is computed under a predefined condition or subset (e.g., genes or cells whose expression exceeds a threshold t), allowing the variability to be assessed within a controlled range.

Supplementary Figures

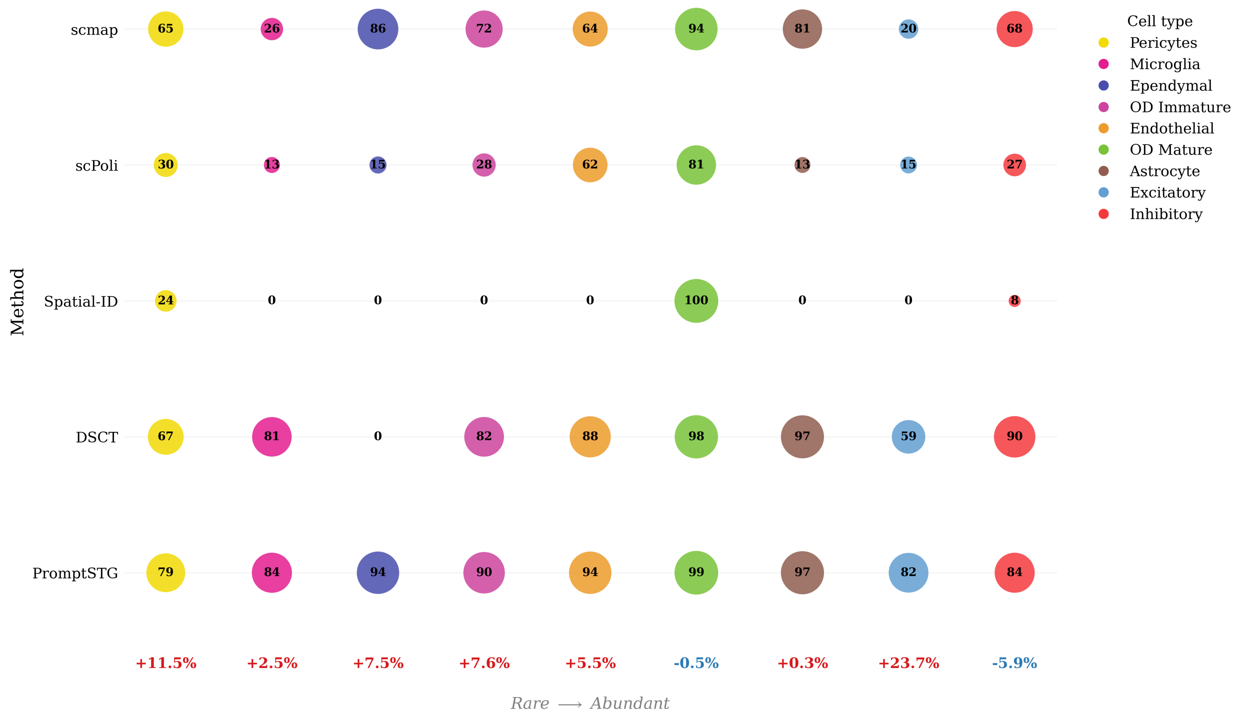

Supplementary Figure 2. Cell type-specific classification accuracy across varying abundance levels. Bubble size represents prediction accuracy, with larger bubbles indicating higher accuracy. Cell types are arranged from left to right in order of decreasing rarity, with the rarest cell types on the left. The bottom panel highlights the performance gain of our model over the best-performing baseline for each cell type.

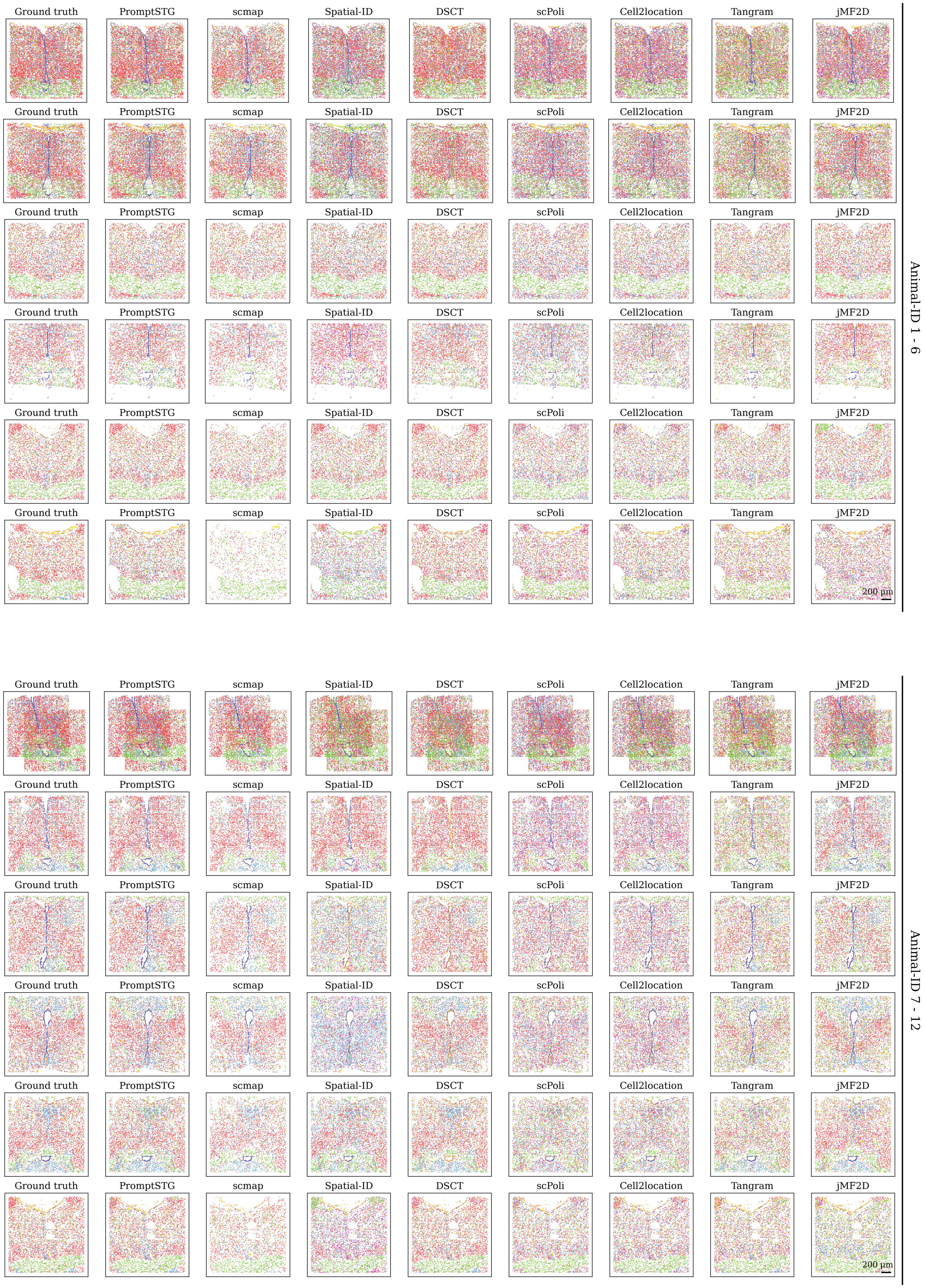
Ground truth
PromptSTG
scmap
Spatial-ID
DSCT
scPoli
Cell2location
Tangram
jMF2D
200 μm
Animal-ID 1 - 6
Ground truth
PromptSTG
scmap
Spatial-ID
DSCT
scPoli
Cell2location
Tangram
jMF2D
200 μm
Animal-ID 7 - 12

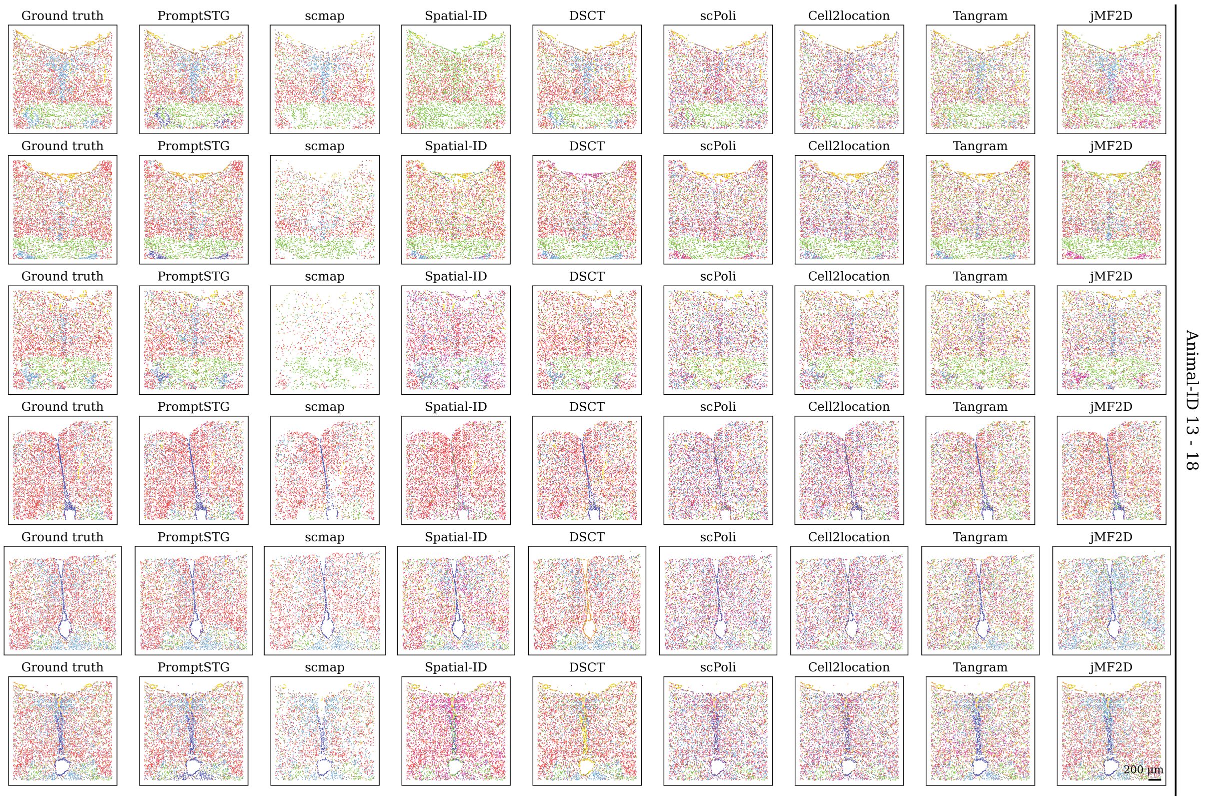
Ground truth
PromptSTG
scmap
Spatial-ID
DSCT
scPoli
Cell2location
Tangram
jMF2D
200 μm
Animal-ID 13 - 18

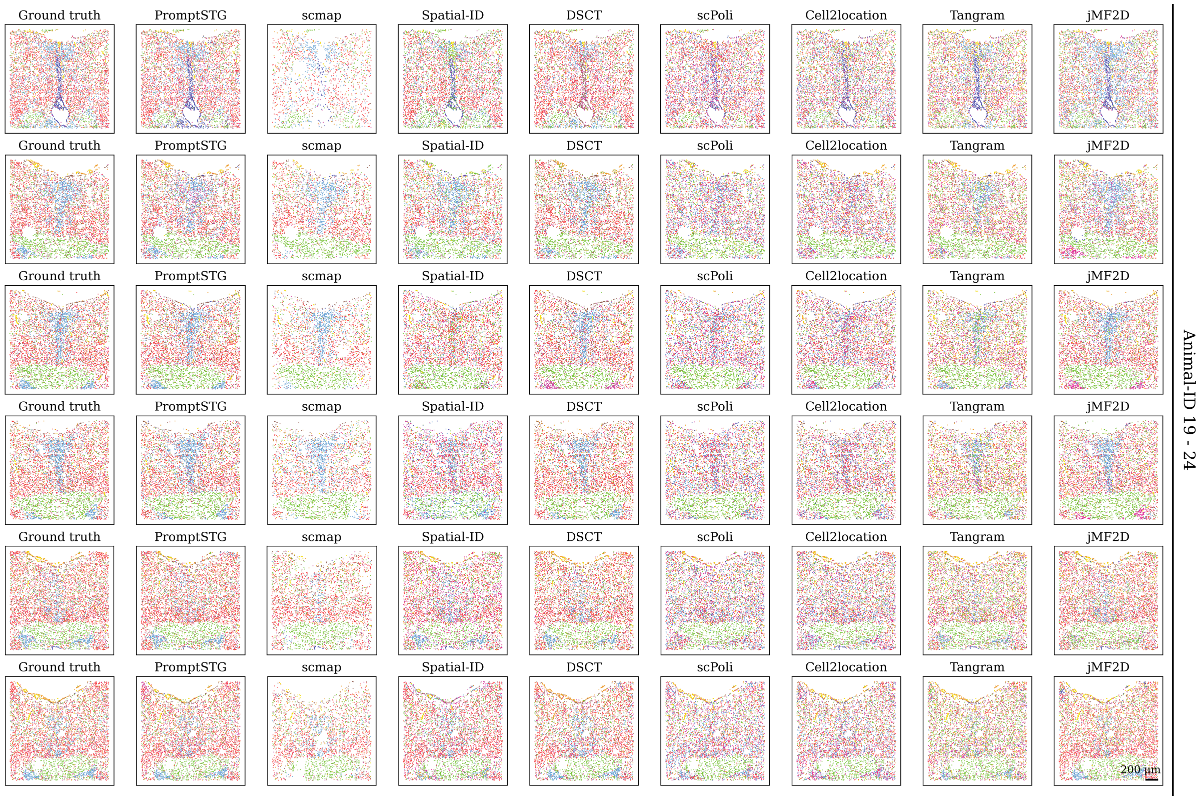
Ground truth
PromptSTG
scmap
Spatial-ID
DSCT
scPoli
Cell2location
Tangram
jMF2D
200 μm
Animal-ID 19 - 24

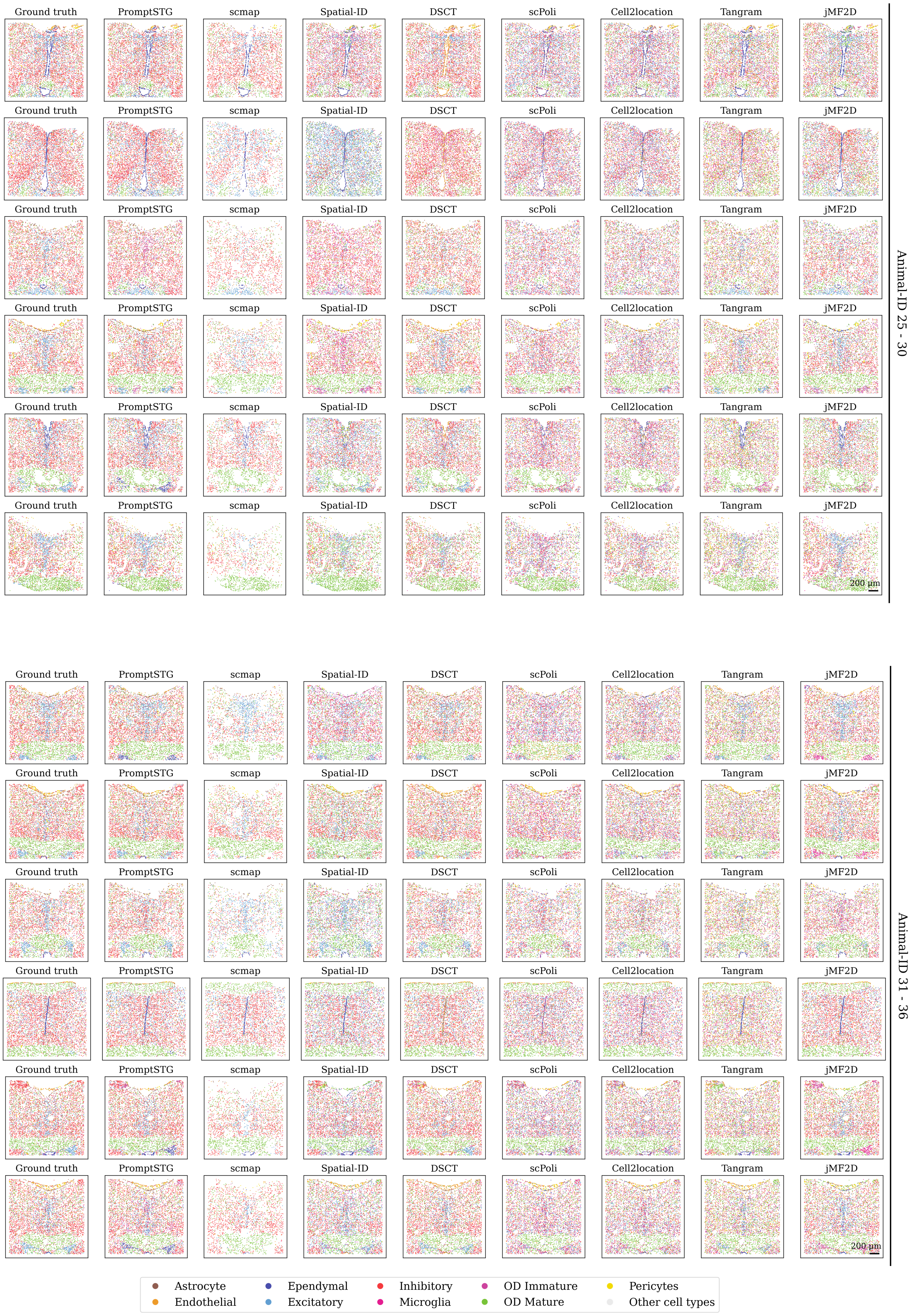

Supplementary Figure 3. Scatter plot comparison of different methods across 36 Animal-ID slices.

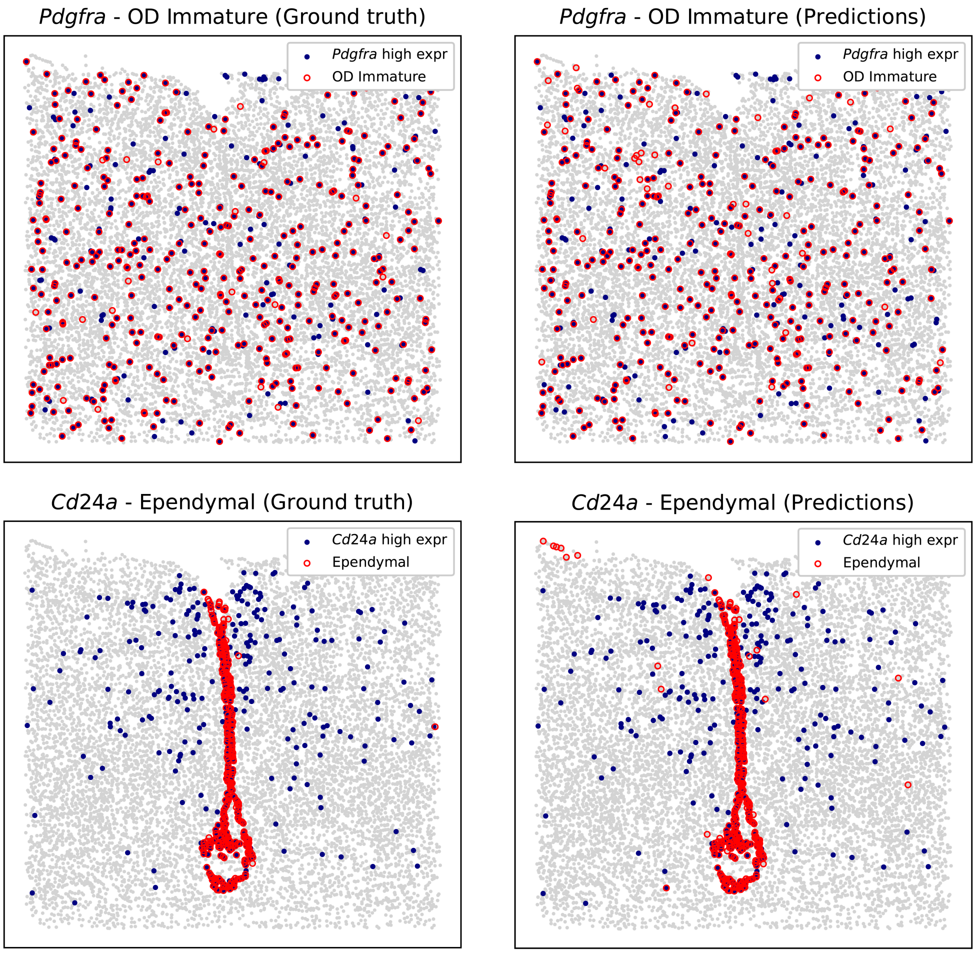

Supplementary Figure 4. Spatial validation of predicted OD Immature and Ependymal cells using marker gene expression. We denote cells within the top 95% of expression for the given gene with solid blue dots, and cells belonging to the specified cell type with red hollow circles.

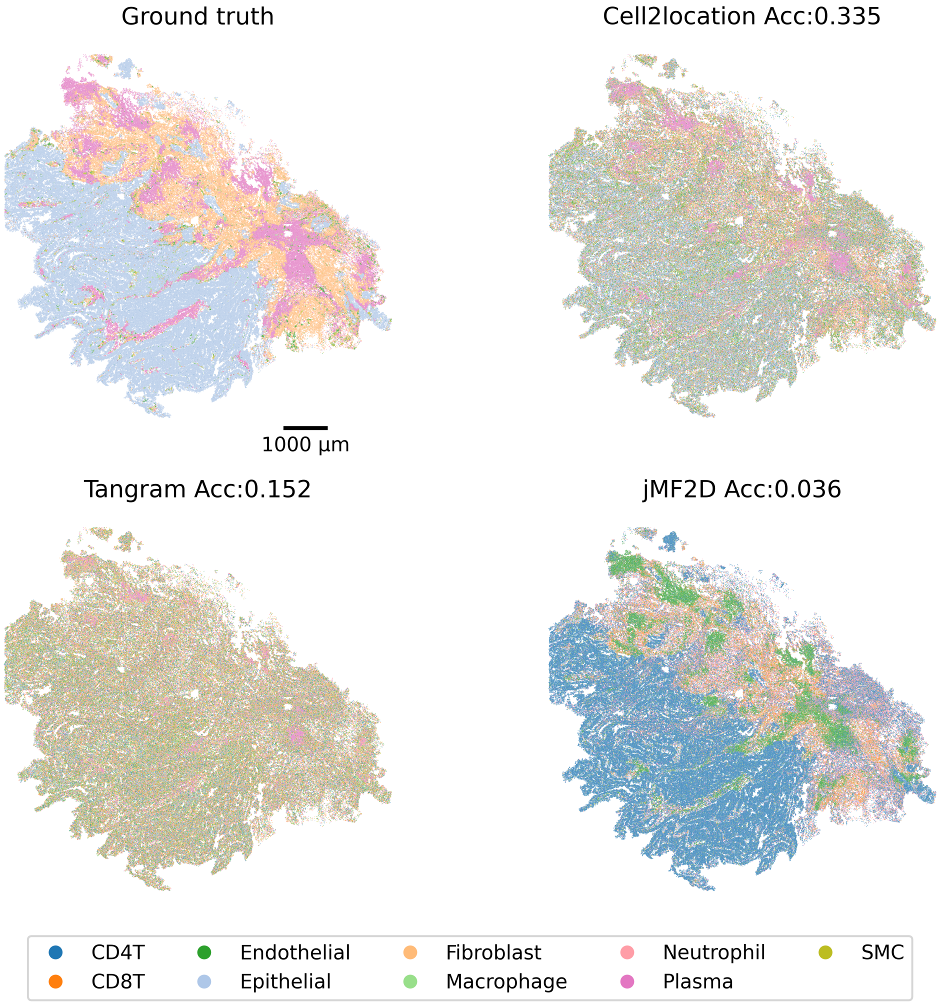

Supplementary Figure 5. Spatial visualization of cell type annotations in the Stereo-seq COAD dataset across Cell2location, Tangram, and jMF2D.

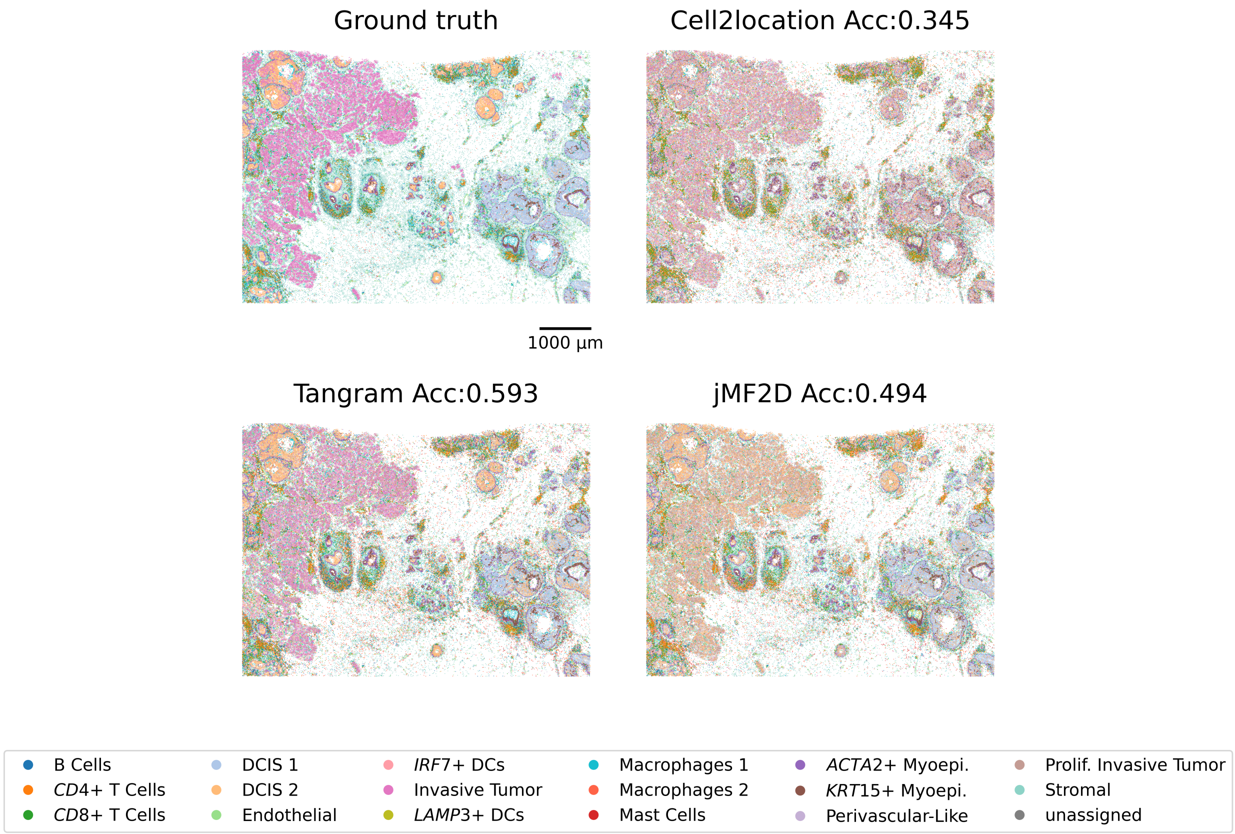

Supplementary Figure 6. Spatial visualization of cell type annotations in the breast cancer Xenium dataset across Cell2location, Tangram, and jMF2D.

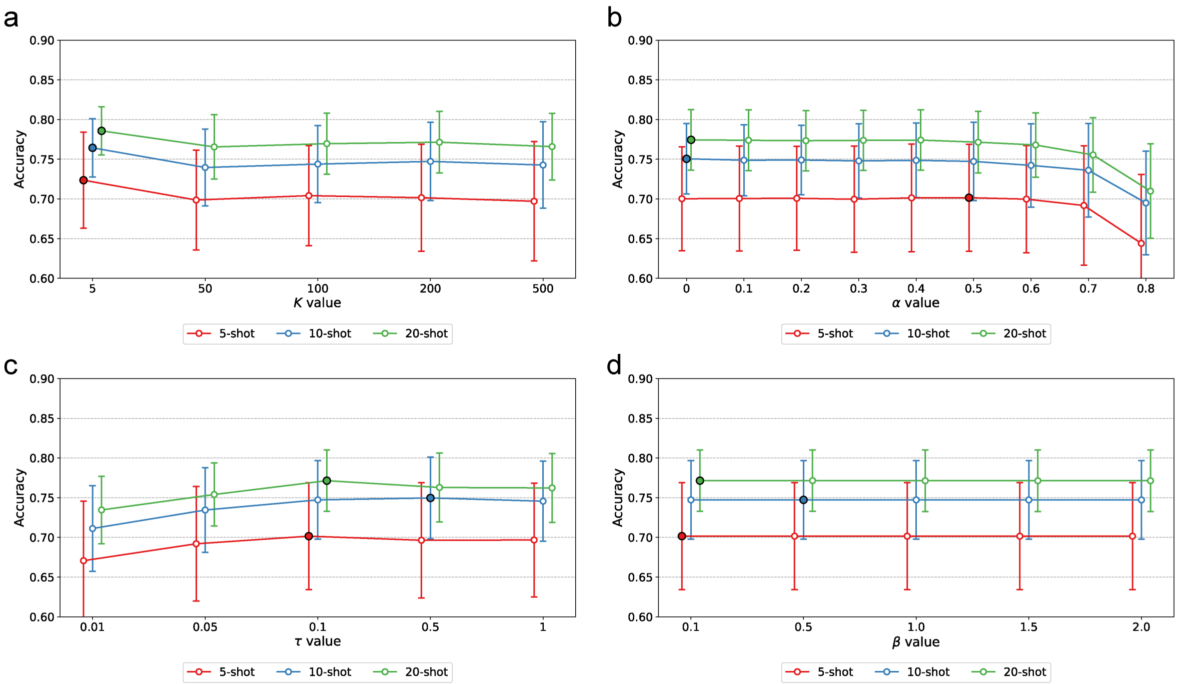

Supplementary Figure 7. Parameter sensitivity results under fixed training epochs. (**a**) Sensitivity analysis of the number of neighbors K. (**b**) Sensitivity analysis of the graph fusion threshold α. (**c**) Sensitivity analysis of the temperature parameter τ. (**d**) Sensitivity analysis of the prototype distance threshold β.

Supplementary Tables

Supplementary Table 2. Summary of cell and gene numbers in the preprocessed datasets.

Dataset	Cells	Genes	Sections
Mouse hypothalamus	874,768	160	36
Colorectal adenocarcinoma (COAD)	324,263	1,425	–
Breast cancer	147,609	313	–

Supplementary Table 3. Accuracy comparison on 36 mouse hypothalamus sections (MERFISH).

Method	5	10	20
Spatial-ID	0.206 ± 0.045	0.328 ± 0.055	0.461 ± 0.044
DSCT	0.635 ± 0.042	0.693 ± 0.044	0.731 ± 0.041
scPoli	0.160 ± 0.017	0.221 ± 0.023	0.365 ± 0.025
scmap	0.371 ± 0.120	0.479 ± 0.115	0.518 ± 0.117
jMF2D	0.529 ± 0.045	0.544 ± 0.074	0.576 ± 0.070
Cell2location	0.270 ± 0.010	0.344 ± 0.009	0.416 ± 0.013
Tangram	0.445 ± 0.036	0.495 ± 0.038	0.512 ± 0.038
PromptSTG	**0.711 ± 0.047**	**0.748 ± 0.038**	**0.764 ± 0.037**

Detailed results are provided in the supplementary file `brain_prediction_detail.pdf`.

Supplementary Table 4. Accuracy comparison on the colorectal adenocarcinoma dataset (COAD, Stereo-seq).

Method	5	10	20
Spatial-ID	0.155 ± 0.201	0.167 ± 0.172	0.333 ± 0.121
DSCT	0.383 ± 0.082	0.451 ± 0.045	0.483 ± 0.044
scPoli	0.191 ± 0.090	0.409 ± 0.154	0.595 ± 0.149
scmap	0.308 ± 0.143	0.446 ± 0.109	0.468 ± 0.075
jMF2D	0.062 ± 0.038	0.060 ± 0.025	0.066 ± 0.026
Cell2location	0.255 ± 0.019	0.258 ± 0.025	0.330 ± 0.016
Tangram	0.144 ± 0.007	0.146 ± 0.003	0.150 ± 0.003
PromptSTG	**0.680 ± 0.071**	**0.715 ± 0.019**	**0.741 ± 0.022**

Supplementary Table 5. Accuracy comparison on the human breast cancer dataset (Xenium).

Method	5	10	20
Spatial-ID	0.108 ± 0.100	0.263 ± 0.096	0.313 ± 0.088
DSCT	0.540 ± 0.056	0.599 ± 0.033	0.651 ± 0.014
scPoli	0.170 ± 0.007	0.238 ± 0.031	0.342 ± 0.024
scmap	0.380 ± 0.040	0.436 ± 0.033	0.467 ± 0.042
jMF2D	0.573 ± 0.047	0.605 ± 0.046	0.588 ± 0.058
Cell2location	0.266 ± 0.026	0.308 ± 0.019	0.337 ± 0.010
Tangram	0.529 ± 0.021	0.564 ± 0.008	0.589 ± 0.011
PromptSTG	**0.665 ± 0.072**	**0.697 ± 0.052**	**0.724 ± 0.045**
