# Supplementary material for: PromptSTG: prototype-guided prompting for few-shot spatial transcriptomics annotation
Source: Brief Bioinform. 2026 Jul 29;27(4):bbag401. doi: 10.1093/bib/bbag401 (PMC13418865; doi:10.1093/bib/bbag401)
Supplement: brain_prediction_detail_bbag401 [file brain_prediction_detail_bbag401.pdf]

mid	PromptSTG-5-shot	PromptSTG-10-shot	PromptSTG-20-shot	DSCT-5-shot	DSCT-10-shot	DSCT-20-shot	Spatial-ID-5-shot	Spatial-ID-10-shot	Spatial-ID-20-shot	Tangram-5-shot	Tangram-10-shot	Tangram-20-shot	Cell2location-5-shot	Cell2location-10-shot	Cell2location-20-shot
1	0.7705 ± 0.0080	0.8092 ± 0.0039	0.8283 ± 0.0207	0.6648 ± 0.0740	0.7412 ± 0.0320	0.7875 ± 0.0127	0.2026 ± 0.1375	0.4178 ± 0.1061	0.4611 ± 0.0987	0.4675 ± 0.0015	0.5168 ± 0.0098	0.5345 ± 0.0068	0.2750 ± 0.0146	0.3437 ± 0.0089	0.4209 ± 0.0057
2	0.7876 ± 0.0091	0.8132 ± 0.0131	0.8223 ± 0.0053	0.6597 ± 0.0228	0.6911 ± 0.0473	0.7357 ± 0.0090	0.1988 ± 0.0261	0.3297 ± 0.0484	0.4829 ± 0.0806	0.4646 ± 0.0008	0.5459 ± 0.0201	0.5713 ± 0.0048	0.2767 ± 0.0161	0.3528 ± 0.0184	0.4254 ± 0.0041
3	0.7570 ± 0.0171	0.7378 ± 0.0192	0.7928 ± 0.0008	0.6918 ± 0.0597	0.7798 ± 0.0199	0.8030 ± 0.0185	0.1469 ± 0.0858	0.3585 ± 0.0535	0.5241 ± 0.1402	0.5223 ± 0.0201	0.5575 ± 0.0032	0.5719 ± 0.0062	0.2823 ± 0.0100	0.3402 ± 0.0027	0.4098 ± 0.0011
4	0.7251 ± 0.0937	0.7420 ± 0.0609	0.8029 ± 0.0043	0.6035 ± 0.0814	0.6765 ± 0.0321	0.7253 ± 0.0368	0.2147 ± 0.1385	0.3407 ± 0.0771	0.4578 ± 0.1051	0.4000 ± 0.0080	0.4457 ± 0.0024	0.4524 ± 0.0065	0.2586 ± 0.0055	0.3283 ± 0.0056	0.4094 ± 0.0034
5	0.7241 ± 0.0051	0.7397 ± 0.0028	0.7795 ± 0.0237	0.6820 ± 0.0470	0.7646 ± 0.0063	0.7830 ± 0.0053	0.1215 ± 0.0734	0.3397 ± 0.1069	0.3749 ± 0.1111	0.5165 ± 0.0191	0.5564 ± 0.0066	0.5630 ± 0.0022	0.2792 ± 0.0067	0.3379 ± 0.0018	0.4057 ± 0.0087
6	0.6082 ± 0.0140	0.6793 ± 0.0380	0.6966 ± 0.0058	0.6191 ± 0.0512	0.6937 ± 0.0303	0.7515 ± 0.0123	0.1583 ± 0.0718	0.2866 ± 0.0928	0.4453 ± 0.0688	0.4635 ± 0.0027	0.5213 ± 0.0081	0.5307 ± 0.0073	0.2536 ± 0.0046	0.3221 ± 0.0067	0.3914 ± 0.0086
7	0.7019 ± 0.0138	0.7598 ± 0.0344	0.7926 ± 0.0344	0.5904 ± 0.0332	0.6398 ± 0.0539	0.6831 ± 0.0519	0.2019 ± 0.0343	0.2831 ± 0.0472	0.4139 ± 0.1115	0.4413 ± 0.0095	0.4938 ± 0.0089	0.5112 ± 0.0023	0.2808 ± 0.0019	0.3554 ± 0.0006	0.4307 ± 0.0015
8	0.8110 ± 0.0087	0.8113 ± 0.0203	0.8221 ± 0.0010	0.7059 ± 0.0274	0.7115 ± 0.0270	0.7418 ± 0.0101	0.2675 ± 0.0870	0.3439 ± 0.0719	0.4946 ± 0.1213	0.3982 ± 0.0220	0.4442 ± 0.0018	0.4643 ± 0.0017	0.2802 ± 0.0095	0.3573 ± 0.0093	0.4402 ± 0.0091
9	0.6785 ± 0.0729	0.7499 ± 0.0063	0.7929 ± 0.0187	0.6401 ± 0.0950	0.6837 ± 0.0426	0.7199 ± 0.0144	0.1943 ± 0.0777	0.2664 ± 0.0529	0.3906 ± 0.0262	0.4830 ± 0.0267	0.5505 ± 0.0104	0.5578 ± 0.0045	0.2730 ± 0.0065	0.3623 ± 0.0018	0.4524 ± 0.0132
10	0.7143 ± 0.0821	0.8108 ± 0.0262	0.8086 ± 0.0033	0.6430 ± 0.0855	0.7217 ± 0.0530	0.7651 ± 0.0302	0.2075 ± 0.0576	0.4445 ± 0.0968	0.4751 ± 0.1213	0.4546 ± 0.0298	0.5061 ± 0.0009	0.5293 ± 0.0072	0.2747 ± 0.0093	0.3450 ± 0.0086	0.4370 ± 0.0084
11	0.7087 ± 0.0557	0.7988 ± 0.0079	0.7891 ± 0.0064	0.6405 ± 0.0654	0.7254 ± 0.0172	0.7477 ± 0.0169	0.2603 ± 0.0540	0.3757 ± 0.0526	0.4686 ± 0.1448	0.4720 ± 0.0178	0.5189 ± 0.0139	0.5436 ± 0.0029	0.2767 ± 0.0086	0.3553 ± 0.0044	0.4356 ± 0.0037
12	0.6748 ± 0.0572	0.7111 ± 0.0308	0.7161 ± 0.0228	0.6493 ± 0.0193	0.7298 ± 0.0170	0.7632 ± 0.0176	0.1495 ± 0.1032	0.3027 ± 0.0897	0.3705 ± 0.0585	0.5048 ± 0.0140	0.5337 ± 0.0192	0.5343 ± 0.0134	0.2772 ± 0.0163	0.3475 ± 0.0105	0.4069 ± 0.0057
13	0.7552 ± 0.0060	0.7631 ± 0.0171	0.7738 ± 0.0090	0.6881 ± 0.1004	0.7540 ± 0.0358	0.7949 ± 0.0142	0.1848 ± 0.1272	0.3712 ± 0.0226	0.4584 ± 0.0199	0.4507 ± 0.0043	0.5135 ± 0.0074	0.5495 ± 0.0178	0.2741 ± 0.0034	0.3487 ± 0.0031	0.4173 ± 0.0082
14	0.7418 ± 0.0072	0.7485 ± 0.0122	0.7518 ± 0.0101	0.5870 ± 0.0856	0.6640 ± 0.0405	0.7159 ± 0.0267	0.2696 ± 0.0857	0.3455 ± 0.1035	0.5542 ± 0.0386	0.4798 ± 0.0048	0.5164 ± 0.0021	0.5454 ± 0.0080	0.2729 ± 0.0143	0.3449 ± 0.0070	0.4079 ± 0.0049
15	0.6591 ± 0.0305	0.7347 ± 0.0228	0.7281 ± 0.0194	0.5669 ± 0.0352	0.6034 ± 0.0523	0.6653 ± 0.0227	0.2451 ± 0.0605	0.2622 ± 0.0290	0.4702 ± 0.0012	0.4129 ± 0.0095	0.4670 ± 0.0177	0.4751 ± 0.0033	0.2542 ± 0.0052	0.3284 ± 0.0014	0.4067 ± 0.0010
16	0.6259 ± 0.0836	0.6685 ± 0.0551	0.7242 ± 0.0123	0.6153 ± 0.0267	0.6543 ± 0.0203	0.6687 ± 0.0435	0.1971 ± 0.1543	0.3171 ± 0.0912	0.4833 ± 0.0488	0.4047 ± 0.0249	0.4592 ± 0.0093	0.4769 ± 0.0046	0.2474 ± 0.0029	0.3326 ± 0.0017	0.4205 ± 0.0052
17	0.7667 ± 0.0237	0.8044 ± 0.0261	0.7967 ± 0.0248	0.6866 ± 0.0334	0.7401 ± 0.0255	0.7683 ± 0.0132	0.2849 ± 0.0194	0.3309 ± 0.0986	0.5344 ± 0.1246	0.4713 ± 0.0125	0.5131 ± 0.0055	0.5373 ± 0.0020	0.2931 ± 0.0049	0.3518 ± 0.0065	0.4148 ± 0.0062
18	0.7493 ± 0.0214	0.7586 ± 0.0207	0.7645 ± 0.0076	0.6358 ± 0.0735	0.7225 ± 0.0200	0.7490 ± 0.0279	0.2288 ± 0.0714	0.3286 ± 0.1246	0.4669 ± 0.0678	0.4676 ± 0.0227	0.5194 ± 0.0047	0.5416 ± 0.0134	0.2762 ± 0.0018	0.3464 ± 0.0069	0.4106 ± 0.0062
19	0.6957 ± 0.0055	0.7230 ± 0.0136	0.7418 ± 0.0145	0.6440 ± 0.0186	0.6925 ± 0.0142	0.7126 ± 0.0206	0.2295 ± 0.0852	0.2580 ± 0.1096	0.4180 ± 0.1452	0.4270 ± 0.0211	0.4781 ± 0.0064	0.4858 ± 0.0048	0.2692 ± 0.0066	0.3489 ± 0.0034	0.4239 ± 0.0029
20	0.6513 ± 0.0153	0.7551 ± 0.0103	0.7490 ± 0.0052	0.6644 ± 0.0169	0.7075 ± 0.0102	0.7316 ± 0.0286	0.2695 ± 0.0699	0.3483 ± 0.0857	0.4480 ± 0.0873	0.4746 ± 0.0344	0.5424 ± 0.0159	0.5597 ± 0.0022	0.2659 ± 0.0036	0.3457 ± 0.0027	0.4175 ± 0.0013
21	0.7429 ± 0.0642	0.7610 ± 0.0426	0.7944 ± 0.0308	0.6251 ± 0.0894	0.7019 ± 0.0416	0.7407 ± 0.0234	0.1904 ± 0.1157	0.3369 ± 0.1163	0.4601 ± 0.0664	0.4426 ± 0.0134	0.4902 ± 0.0078	0.5146 ± 0.0054	0.2636 ± 0.0018	0.3434 ± 0.0073	0.4200 ± 0.0068
22	0.6693 ± 0.0658	0.7269 ± 0.0045	0.7617 ± 0.0096	0.5986 ± 0.0553	0.6744 ± 0.0187	0.7214 ± 0.0216	0.1171 ± 0.0144	0.2924 ± 0.1032	0.4850 ± 0.0106	0.4330 ± 0.0254	0.4883 ± 0.0104	0.5110 ± 0.0033	0.2702 ± 0.0046	0.3447 ± 0.0068	0.4215 ± 0.0113
23	0.7606 ± 0.0029	0.7745 ± 0.0103	0.7716 ± 0.0087	0.6808 ± 0.0371	0.7093 ± 0.0161	0.7594 ± 0.0302	0.1570 ± 0.0745	0.3111 ± 0.0570	0.5227 ± 0.0316	0.4848 ± 0.0191	0.5133 ± 0.0139	0.5313 ± 0.0038	0.2823 ± 0.0029	0.3457 ± 0.0068	0.4219 ± 0.0066
24	0.7084 ± 0.0109	0.7529 ± 0.0243	0.7815 ± 0.0074	0.6370 ± 0.0262	0.7276 ± 0.0337	0.7609 ± 0.0316	0.2487 ± 0.0720	0.3612 ± 0.1077	0.5173 ± 0.0825	0.4716 ± 0.0100	0.5226 ± 0.0121	0.5231 ± 0.0092	0.2688 ± 0.0053	0.3531 ± 0.0119	0.4178 ± 0.0070
25	0.7428 ± 0.0119	0.7644 ± 0.0049	0.7410 ± 0.0161	0.6565 ± 0.0764	0.7048 ± 0.0363	0.7402 ± 0.0094	0.2220 ± 0.1043	0.4090 ± 0.1097	0.4532 ± 0.1889	0.4485 ± 0.0326	0.4867 ± 0.0031	0.5046 ± 0.0088	0.2664 ± 0.0091	0.3385 ± 0.0077	0.4063 ± 0.0052
26	0.7162 ± 0.0824	0.7730 ± 0.0429	0.8008 ± 0.0138	0.5401 ± 0.0494	0.5821 ± 0.0271	0.5993 ± 0.0143	0.2647 ± 0.0400	0.3465 ± 0.0846	0.4879 ± 0.0352	0.3965 ± 0.0140	0.4189 ± 0.0068	0.4320 ± 0.0044	0.2650 ± 0.0097	0.3479 ± 0.0040	0.4193 ± 0.0114
27	0.6602 ± 0.0520	0.7328 ± 0.0159	0.7566 ± 0.0115	0.5993 ± 0.0525	0.6597 ± 0.0524	0.6937 ± 0.0583	0.1392 ± 0.0867	0.2284 ± 0.0874	0.4552 ± 0.1008	0.4185 ± 0.0106	0.4516 ± 0.0103	0.4527 ± 0.0068	0.2794 ± 0.0067	0.3584 ± 0.0026	0.4132 ± 0.0053
28	0.6864 ± 0.0109	0.7367 ± 0.0251	0.7522 ± 0.0352	0.6658 ± 0.0434	0.6815 ± 0.0501	0.7214 ± 0.0282	0.2001 ± 0.1092	0.2884 ± 0.0535	0.5017 ± 0.0398	0.4546 ± 0.0145	0.5004 ± 0.0081	0.5191 ± 0.0058	0.2651 ± 0.0053	0.3384 ± 0.0044	0.3987 ± 0.0068
29	0.7442 ± 0.0041	0.7641 ± 0.0029	0.7854 ± 0.0046	0.6044 ± 0.0220	0.6376 ± 0.0592	0.7220 ± 0.0215	0.1922 ± 0.1315	0.3808 ± 0.1385	0.5007 ± 0.1108	0.3822 ± 0.0126	0.4233 ± 0.0054	0.4404 ± 0.0086	0.2571 ± 0.0068	0.3297 ± 0.0046	0.3972 ± 0.0035
30	0.6529 ± 0.0420	0.6895 ± 0.0123	0.6850 ± 0.0087	0.5899 ± 0.0501	0.6523 ± 0.0538	0.6968 ± 0.0239	0.1575 ± 0.0957	0.2659 ± 0.0507	0.4462 ± 0.1012	0.4063 ± 0.0089	0.4673 ± 0.0111	0.4934 ± 0.0129	0.2590 ± 0.0076	0.3362 ± 0.0177	0.4021 ± 0.0104
31	0.6900 ± 0.0014	0.7016 ± 0.0296	0.7472 ± 0.0009	0.5905 ± 0.0892	0.6646 ± 0.0185	0.7038 ± 0.0234	0.2475 ± 0.0627	0.3306 ± 0.1062	0.4466 ± 0.0466	0.4282 ± 0.0070	0.4850 ± 0.0085	0.4982 ± 0.0043	0.2669 ± 0.0042	0.3327 ± 0.0037	0.4021 ± 0.0033
32	0.7166 ± 0.0243	0.7445 ± 0.0062	0.7415 ± 0.0294	0.7056 ± 0.0336	0.7575 ± 0.0288	0.7835 ± 0.0156	0.2355 ± 0.0646	0.3877 ± 0.0931	0.4633 ± 0.0998	0.4260 ± 0.0125	0.5073 ± 0.0034	0.5227 ± 0.0087	0.2744 ± 0.0135	0.3388 ± 0.0084	0.4097 ± 0.0024
33	0.7234 ± 0.0149	0.7500 ± 0.0141	0.7501 ± 0.0008	0.6463 ± 0.0309	0.6888 ± 0.0339	0.7164 ± 0.0289	0.2507 ± 0.0690	0.3791 ± 0.1268	0.4127 ± 0.0899	0.4157 ± 0.0086	0.4395 ± 0.0158	0.4670 ± 0.0090	0.2491 ± 0.0098	0.3375 ± 0.0110	0.4133 ± 0.0084
34	0.7205 ± 0.0380	0.7463 ± 0.0329	0.7514 ± 0.0129	0.6737 ± 0.0767	0.6990 ± 0.0564	0.7426 ± 0.0268	0.2085 ± 0.1470	0.3491 ± 0.0626	0.3994 ± 0.1410	0.3832 ± 0.0266	0.4538 ± 0.0197	0.4766 ± 0.0025	0.2722 ± 0.0081	0.3376 ± 0.0113	0.4112 ± 0.0033
35	0.7010 ± 0.0128	0.7164 ± 0.0062	0.7257 ± 0.0084	0.5831 ± 0.0804	0.6522 ± 0.0636	0.7117 ± 0.0204	0.1639 ± 0.1093	0.1646 ± 0.0916	0.4597 ± 0.0938	0.4099 ± 0.0277	0.4594 ± 0.0055	0.4857 ± 0.0076	0.2707 ± 0.0099	0.3473 ± 0.0033	0.4152 ± 0.0031
36	0.6369 ± 0.0894	0.6809 ± 0.0156	0.6904 ± 0.0027	0.5773 ± 0.0377	0.6662 ± 0.0183	0.6963 ± 0.0228	0.2043 ± 0.1099	0.3119 ± 0.0607	0.4080 ± 0.0963	0.4558 ± 0.0316	0.5048 ± 0.0166	0.5096 ± 0.0079	0.2717 ± 0.0119	0.3461 ± 0.0041	0.4147 ± 0.0048
Overall	0.711 ± 0.047	0.748 ± 0.038	0.764 ± 0.037	0.635 ± 0.042	0.693 ± 0.044	0.731 ± 0.041	0.206 ± 0.045	0.328 ± 0.055	0.461 ± 0.044	0.445 ± 0.036	0.495 ± 0.038	0.512 ± 0.038	0.270 ± 0.010	0.344 ± 0.009	0.416 ± 0.013

scmap-5-shot	scmap-10-shot	scmap-20-shot	jMF2D-5-shot	jMF2D-10-shot	jMF2D-20-shot	scPoli-5-shot	scPoli-10-shot	scPoli-20-shot
0.5644 ± 0.0060	0.6572 ± 0.0267	0.6909 ± 0.0068	0.5550 ± 0.1193	0.5118 ± 0.2273	0.5076 ± 0.1755	0.1978 ± 0.0542	0.2315 ± 0.0549	0.3832 ± 0.0284
0.6006 ± 0.0073	0.6854 ± 0.0103	0.7043 ± 0.0085	0.5666 ± 0.0820	0.3980 ± 0.1121	0.6574 ± 0.0104	0.1862 ± 0.0439	0.2547 ± 0.0584	0.3912 ± 0.0455
0.6024 ± 0.0294	0.6842 ± 0.0211	0.7109 ± 0.0136	0.6042 ± 0.0504	0.6271 ± 0.0407	0.5862 ± 0.1045	0.1813 ± 0.0377	0.2582 ± 0.0572	0.3871 ± 0.0399
0.5389 ± 0.0277	0.6264 ± 0.0211	0.6707 ± 0.0364	0.5244 ± 0.0704	0.4218 ± 0.1539	0.5403 ± 0.0687	0.1582 ± 0.0253	0.2176 ± 0.0435	0.3952 ± 0.0236
0.5048 ± 0.0223	0.5726 ± 0.0060	0.6514 ± 0.0170	0.5110 ± 0.1311	0.5514 ± 0.0328	0.4847 ± 0.0992	0.1910 ± 0.0531	0.2471 ± 0.0537	0.3963 ± 0.0434
0.3098 ± 0.0117	0.4617 ± 0.0254	0.4953 ± 0.0096	0.4980 ± 0.1146	0.6171 ± 0.0065	0.5681 ± 0.0509	0.1818 ± 0.0382	0.2468 ± 0.0498	0.3781 ± 0.0470
0.4684 ± 0.0213	0.5836 ± 0.0143	0.6442 ± 0.0262	0.6106 ± 0.0526	0.6496 ± 0.0424	0.6233 ± 0.0489	0.1817 ± 0.0374	0.2300 ± 0.0660	0.3928 ± 0.0335
0.5359 ± 0.0401	0.5599 ± 0.0343	0.6368 ± 0.0037	0.5924 ± 0.0554	0.4684 ± 0.1044	0.5722 ± 0.1284	0.1627 ± 0.0300	0.2540 ± 0.0444	0.4120 ± 0.0409
0.4737 ± 0.0418	0.5713 ± 0.0117	0.5828 ± 0.0071	0.4833 ± 0.0337	0.5164 ± 0.1351	0.5229 ± 0.1539	0.1382 ± 0.0258	0.1933 ± 0.0519	0.3552 ± 0.0490
0.4887 ± 0.0423	0.6213 ± 0.0134	0.6499 ± 0.0045	0.5770 ± 0.0452	0.6403 ± 0.0085	0.6471 ± 0.0502	0.1389 ± 0.0372	0.1990 ± 0.0636	0.3763 ± 0.0257
0.4564 ± 0.0274	0.5178 ± 0.0174	0.5764 ± 0.0167	0.5620 ± 0.0661	0.5204 ± 0.0702	0.4981 ± 0.1002	0.1517 ± 0.0234	0.1987 ± 0.0405	0.3541 ± 0.0435
0.3360 ± 0.0244	0.4306 ± 0.0431	0.5066 ± 0.0061	0.5002 ± 0.0382	0.4918 ± 0.1075	0.4240 ± 0.1336	0.1626 ± 0.0447	0.2006 ± 0.0483	0.3385 ± 0.0345
0.4678 ± 0.0586	0.5606 ± 0.0398	0.6182 ± 0.0108	0.5226 ± 0.0687	0.5353 ± 0.1018	0.6064 ± 0.0563	0.1576 ± 0.0375	0.2438 ± 0.0598	0.3995 ± 0.0500
0.3481 ± 0.0092	0.4801 ± 0.0088	0.4865 ± 0.0276	0.5556 ± 0.0844	0.5785 ± 0.0825	0.5485 ± 0.0928	0.1547 ± 0.0375	0.2244 ± 0.0402	0.3659 ± 0.0584
0.1608 ± 0.0217	0.2212 ± 0.0067	0.2632 ± 0.0224	0.4651 ± 0.0463	0.5404 ± 0.0579	0.5338 ± 0.0299	0.1353 ± 0.0315	0.1959 ± 0.0407	0.3245 ± 0.0430
0.3349 ± 0.0007	0.3434 ± 0.0472	0.3986 ± 0.0451	0.5084 ± 0.0205	0.4414 ± 0.1106	0.5119 ± 0.1288	0.1320 ± 0.0406	0.1748 ± 0.0428	0.3198 ± 0.0519
0.4422 ± 0.0890	0.5807 ± 0.0054	0.6327 ± 0.0243	0.5461 ± 0.0164	0.6176 ± 0.0780	0.6313 ± 0.0509	0.1849 ± 0.0370	0.2575 ± 0.0528	0.3965 ± 0.0559
0.2415 ± 0.0287	0.4806 ± 0.0172	0.4880 ± 0.0105	0.5481 ± 0.0773	0.6277 ± 0.0294	0.6742 ± 0.0182	0.1608 ± 0.0396	0.2257 ± 0.0449	0.3797 ± 0.0512
0.1599 ± 0.0140	0.3136 ± 0.0243	0.3628 ± 0.0076	0.4543 ± 0.0148	0.4760 ± 0.0711	0.5601 ± 0.0577	0.1488 ± 0.0347	0.2013 ± 0.0366	0.3470 ± 0.0438
0.4474 ± 0.0520	0.5423 ± 0.0259	0.5549 ± 0.0171	0.5824 ± 0.0311	0.5121 ± 0.1076	0.4532 ± 0.1129	0.1275 ± 0.0241	0.1848 ± 0.0463	0.3276 ± 0.0326
0.3349 ± 0.0119	0.4615 ± 0.0343	0.4502 ± 0.0275	0.4925 ± 0.0665	0.5991 ± 0.0199	0.6010 ± 0.0902	0.1514 ± 0.0284	0.2047 ± 0.0354	0.3323 ± 0.0332
0.3060 ± 0.0244	0.4635 ± 0.0218	0.4964 ± 0.0352	0.5282 ± 0.0614	0.4342 ± 0.2486	0.6346 ± 0.0540	0.1500 ± 0.0376	0.1786 ± 0.0415	0.3374 ± 0.0434
0.3294 ± 0.0542	0.4558 ± 0.0724	0.5233 ± 0.0362	0.5273 ± 0.0245	0.5095 ± 0.1277	0.5053 ± 0.1062	0.1650 ± 0.0370	0.2271 ± 0.0476	0.3717 ± 0.0342
0.3997 ± 0.0074	0.5268 ± 0.0351	0.5408 ± 0.0323	0.5748 ± 0.0500	0.6442 ± 0.0541	0.6976 ± 0.0185	0.1474 ± 0.0231	0.2245 ± 0.0510	0.3722 ± 0.0624
0.3142 ± 0.0151	0.4208 ± 0.0350	0.4692 ± 0.0247	0.5618 ± 0.0719	0.5665 ± 0.1004	0.5634 ± 0.0621	0.1747 ± 0.0503	0.2207 ± 0.0601	0.3589 ± 0.0498
0.2824 ± 0.0075	0.4706 ± 0.0287	0.4445 ± 0.0308	0.4498 ± 0.0361	0.3911 ± 0.0518	0.5441 ± 0.1223	0.1666 ± 0.0376	0.2576 ± 0.0576	0.3729 ± 0.0478
0.2184 ± 0.0238	0.3660 ± 0.0339	0.3961 ± 0.0093	0.5201 ± 0.1305	0.4484 ± 0.1308	0.5374 ± 0.0115	0.1484 ± 0.0111	0.2356 ± 0.0574	0.3601 ± 0.0594
0.2949 ± 0.0404	0.4131 ± 0.0077	0.4345 ± 0.0112	0.4837 ± 0.0512	0.5828 ± 0.0451	0.6548 ± 0.0254	0.1588 ± 0.0301	0.2201 ± 0.0450	0.3797 ± 0.0451
0.3620 ± 0.0891	0.4558 ± 0.0599	0.5239 ± 0.0024	0.5167 ± 0.0293	0.6236 ± 0.0524	0.6431 ± 0.0156	0.1605 ± 0.0166	0.2477 ± 0.0606	0.3854 ± 0.0458
0.2500 ± 0.0677	0.3481 ± 0.0109	0.3301 ± 0.0182	0.4874 ± 0.0531	0.5847 ± 0.0394	0.6260 ± 0.0065	0.1596 ± 0.0303	0.2060 ± 0.0410	0.3343 ± 0.0466
0.2646 ± 0.0912	0.3909 ± 0.0082	0.3835 ± 0.0415	0.5744 ± 0.0752	0.5922 ± 0.0320	0.4763 ± 0.0628	0.1616 ± 0.0449	0.2156 ± 0.0287	0.3128 ± 0.0288
0.3892 ± 0.0194	0.4452 ± 0.0124	0.5485 ± 0.0177	0.5713 ± 0.0340	0.5102 ± 0.0655	0.6765 ± 0.0080	0.1729 ± 0.0410	0.2235 ± 0.0549	0.3690 ± 0.0518
0.2783 ± 0.0198	0.3579 ± 0.0199	0.3679 ± 0.0273	0.4887 ± 0.0349	0.5856 ± 0.0148	0.5903 ± 0.0435	0.1435 ± 0.0309	0.2048 ± 0.0496	0.3653 ± 0.0445
0.3755 ± 0.0083	0.5652 ± 0.0154	0.6047 ± 0.0130	0.4797 ± 0.1515	0.6118 ± 0.0531	0.5359 ± 0.1068	0.1736 ± 0.0454	0.2231 ± 0.0564	0.3529 ± 0.0287
0.2282 ± 0.0300	0.3392 ± 0.0725	0.3867 ± 0.0079	0.5758 ± 0.0074	0.6103 ± 0.0415	0.6814 ± 0.0071	0.1457 ± 0.0388	0.2140 ± 0.0385	0.3474 ± 0.0508
0.2632 ± 0.0625	0.2824 ± 0.0263	0.4148 ± 0.0050	0.4621 ± 0.1034	0.5639 ± 0.0170	0.6256 ± 0.0261	0.1399 ± 0.0429	0.2126 ± 0.0565	0.3570 ± 0.0582
0.371 ± 0.120	0.479 ± 0.115	0.518 ± 0.117	0.529 ± 0.045	0.544 ± 0.074	0.576 ± 0.070	0.160 ± 0.017	0.221 ± 0.023	0.365 ± 0.025
